# Correction: Untargeted lipidomics reveals unique lipid signatures of extracellular vesicles from porcine colostrum and milk

**DOI:** 10.1371/journal.pone.0326133

**Published:** 2025-06-09

**Authors:** Rafaela Furioso Ferreira, Morteza H. Ghaffari, Fabrizio Ceciliani, Manuela Fontana, Donatella Caruso, Matteo Audano, Giovanni Savoini, Alessandro Agazzi, Vladimir Mrljak, Helga Sauerwein

The following Acknowledgments is missing from the published article [1]: This publication was supported by the Open Access Publication Fund of the University of Bonn.
