# Supplementary material for: Untargeted lipidomics reveals unique lipid signatures of extracellular vesicles from porcine colostrum and milk
Source: PLoS One. 2025 Feb 13;20(2):e0313683. doi: 10.1371/journal.pone.0313683 (PMC11825007; doi:10.1371/journal.pone.0313683)
Supplement: S1 Fig — https://doi.org/10.6084/m9.figshare.28016336.v2. (PDF) [file pone.0313683.s001.pdf]

A

QC sample chromatogram (pool) acquired in positive polarity

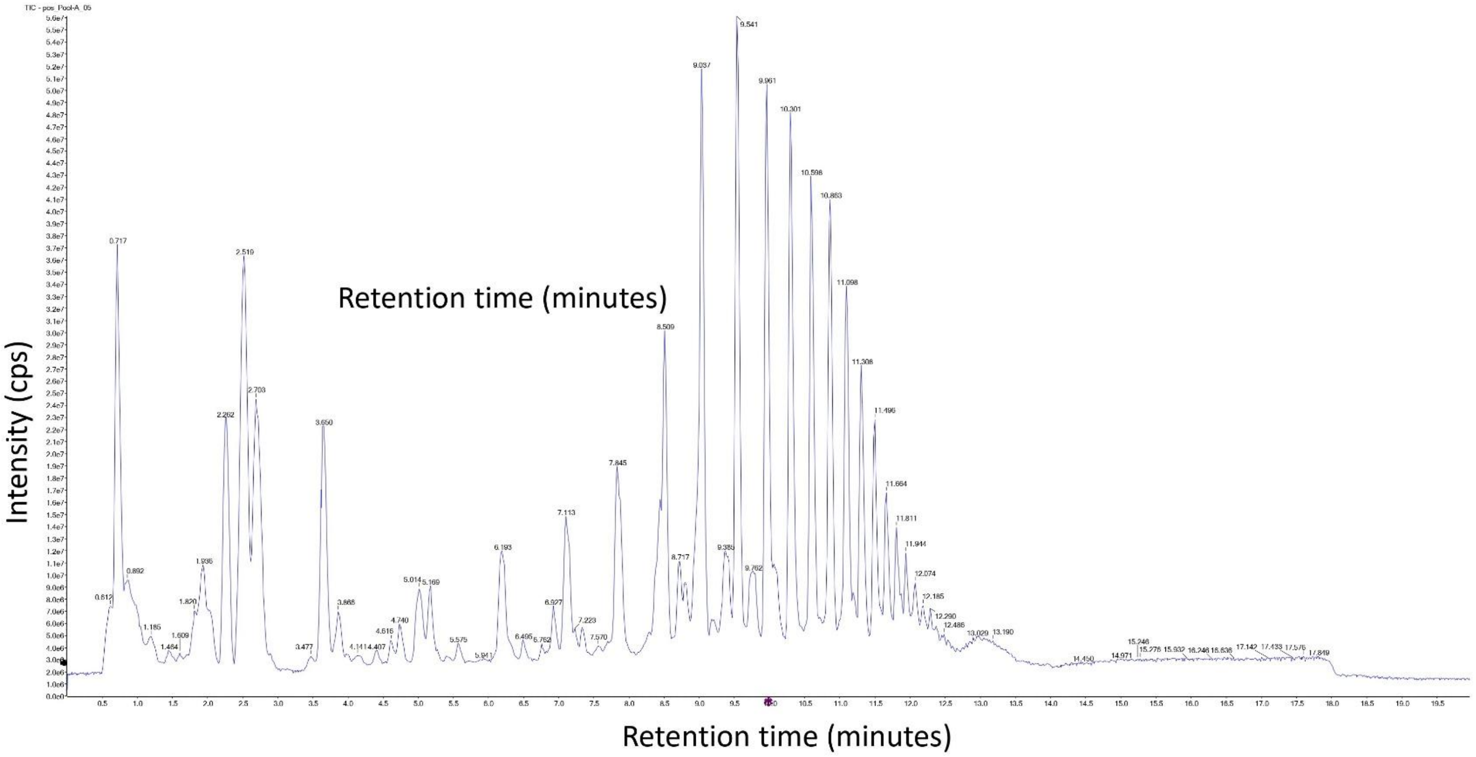

# B

QC sample chromatogram (pool) acquired in positive polarity

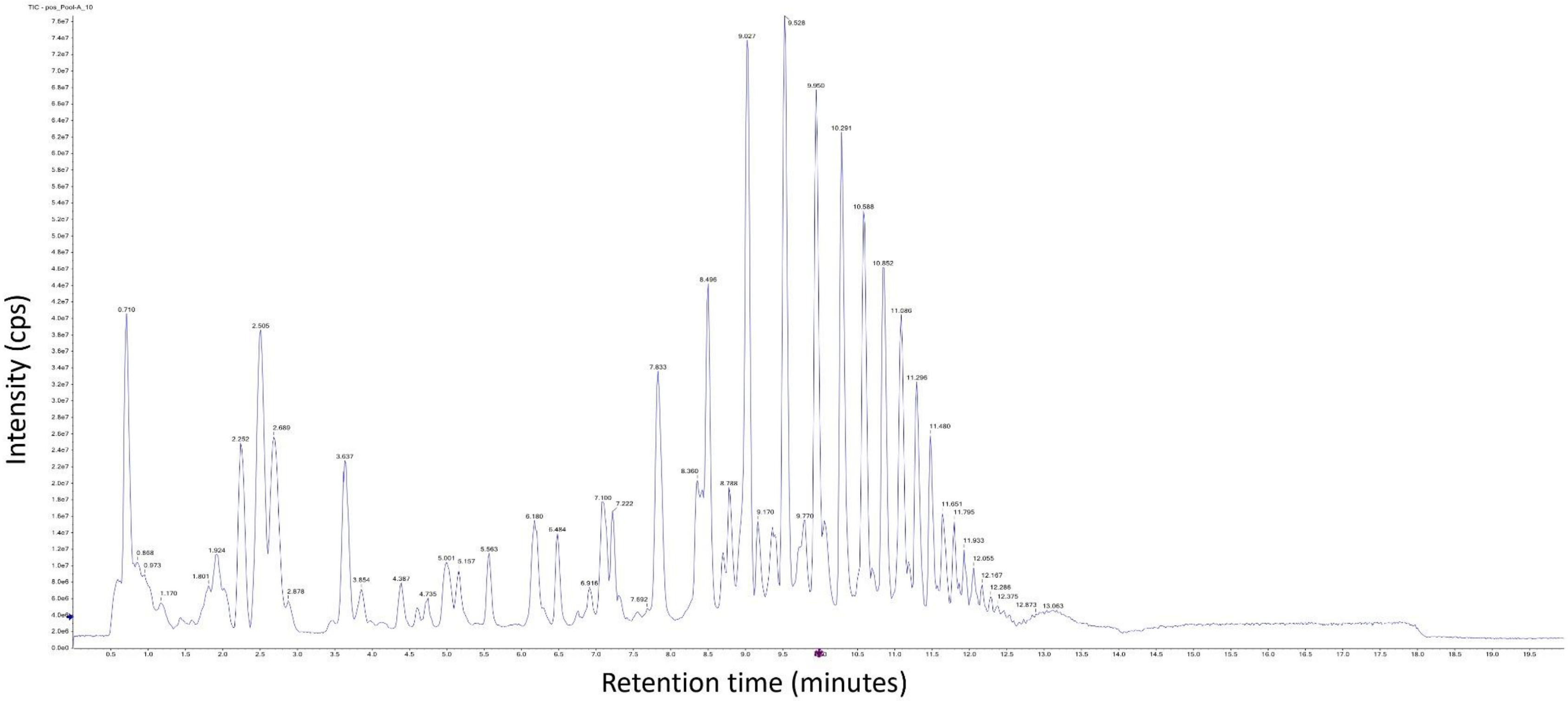

C

QC sample chromatogram (pool) acquired in negative polarity

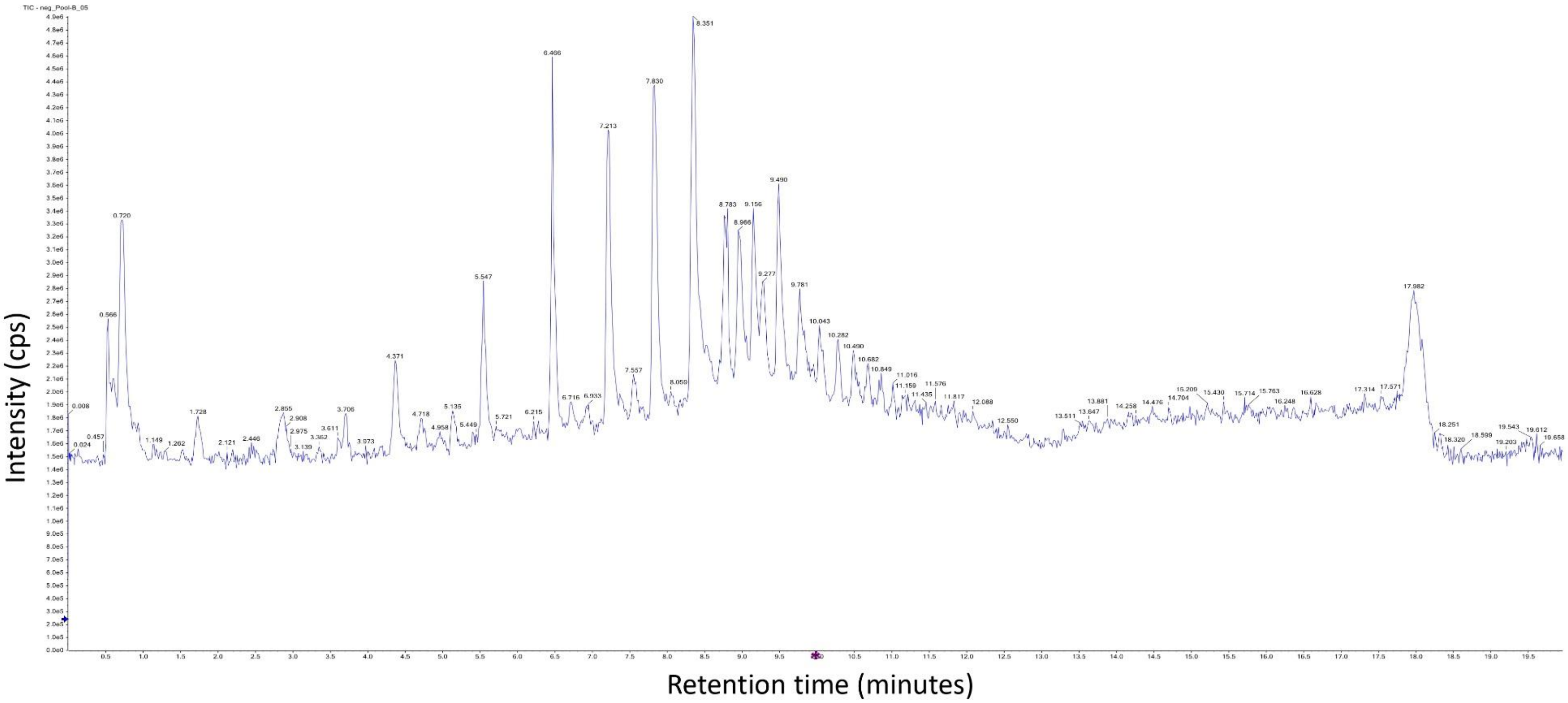

D

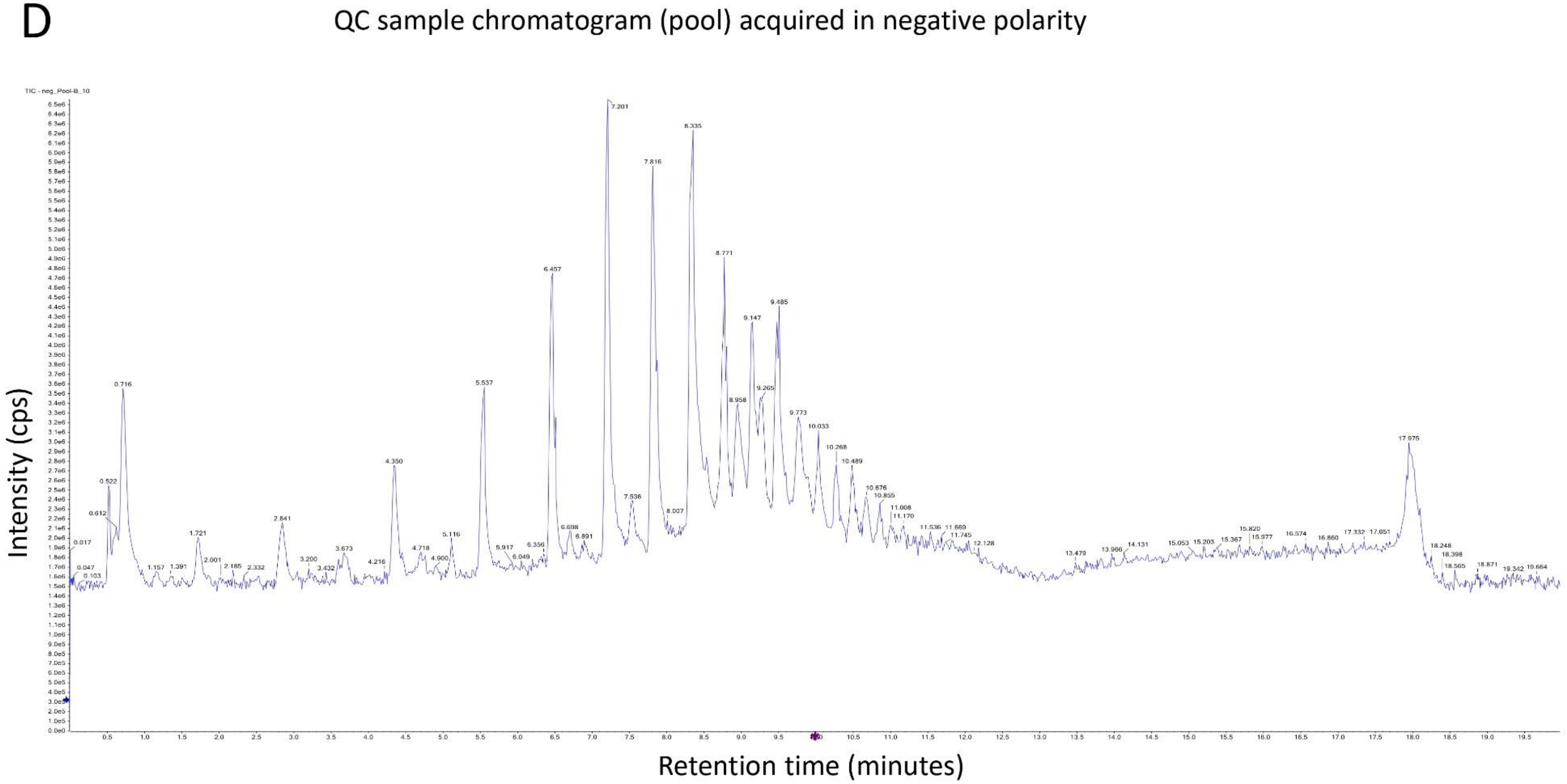


**S1 Fig.** shows representative ion chromatograms from QC samples acquired in positive (Fig. S3A, S3B) and negative (Fig. S3C, S3D) ionization modes, which were analyzed to ensure data quality and consistency throughout the study.
