# Supplementary material for: Untargeted lipidomics reveals unique lipid signatures of extracellular vesicles from porcine colostrum and milk
Source: PLoS One. 2025 Feb 13;20(2):e0313683. doi: 10.1371/journal.pone.0313683 (PMC11825007; doi:10.1371/journal.pone.0313683)
Supplement: S2 Fig — https://doi.org/10.6084/m9.figshare.28016378.v1. (PDF) [file pone.0313683.s002.pdf]

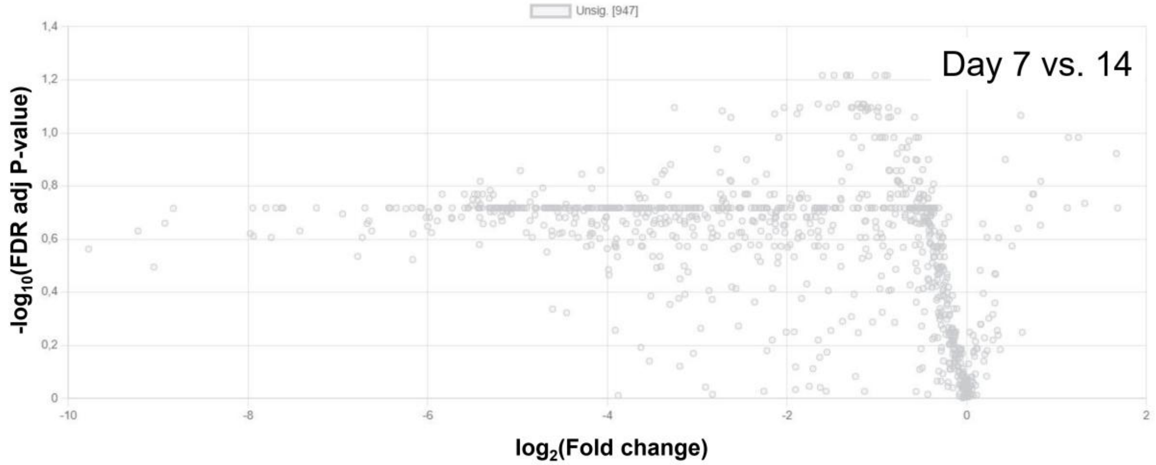

S2 Fig. Volcano plot of $\log_2$ fold changes (x-axis) and their associated $-\log_{10}$ FDR adjusted p-values (y-axis) of all identified lipids in the comparison between milk exosomes at day 7 and milk exosomes at day 14. No significantly different lipids were found (t-test FDR adjusted p-value threshold at 0.05, fold change threshold at 2).
