# Supplementary material for: Untargeted lipidomics reveals unique lipid signatures of extracellular vesicles from porcine colostrum and milk
Source: PLoS One. 2025 Feb 13;20(2):e0313683. doi: 10.1371/journal.pone.0313683 (PMC11825007; doi:10.1371/journal.pone.0313683)
Supplement: S3 Fig — https://doi.org/10.6084/m9.figshare.28016396.v1. (PDF) [file pone.0313683.s003.pdf]

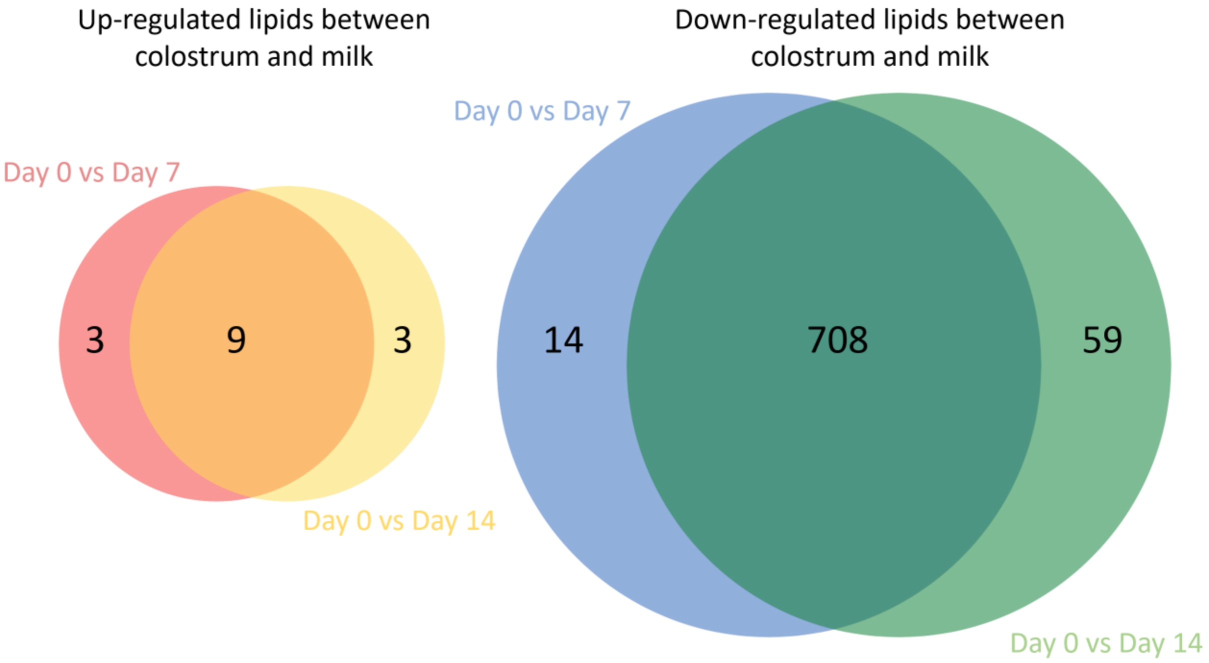

S3 Fig. Venn diagram representing the number of differentially expressed lipids elements found in comparison between colostrum exosomes (day 0) and milk exosomes at day 7 and 14, featuring that most of lipid elements were shared between comparisons.
