# Supplementary material for: Untargeted lipidomics reveals unique lipid signatures of extracellular vesicles from porcine colostrum and milk
Source: PLoS One. 2025 Feb 13;20(2):e0313683. doi: 10.1371/journal.pone.0313683 (PMC11825007; doi:10.1371/journal.pone.0313683)

S4 Fig. Western blotting (WB) of EV marker TSG 101 in samples from both groups after ultracentrifugation coupled with size exclusion chromatography (SEC). Fractions 2 and 3 from SEC contained EV and were pooled together for further analysis. Imaging was performed with a VersaDoc MP4000 imaging system (Bio-Rad, Hercules, CA, USA). Original images showing full the length membrane with different exposure levels are presented.

Lane/Gel 1	Sample/Volume
1	------- 4uL pp
2	Marker 8uL
3	------- 4uL pp
4	6 Fraction 1 24uL GS Day 0
5	6 Fraction 2 24uL GS Day 0
6	6 Fraction 3 24uL GS Day 0
7	6 Fraction 4 24uL GS Day 0
8	9 Fraction 2 24uL GL Day 0
9	9 Fraction 3 24uL GL Day 0
10	8 Fraction 2 24uL GS Day 7
11	8 Fraction 3 24uL GS Day 7
12	------

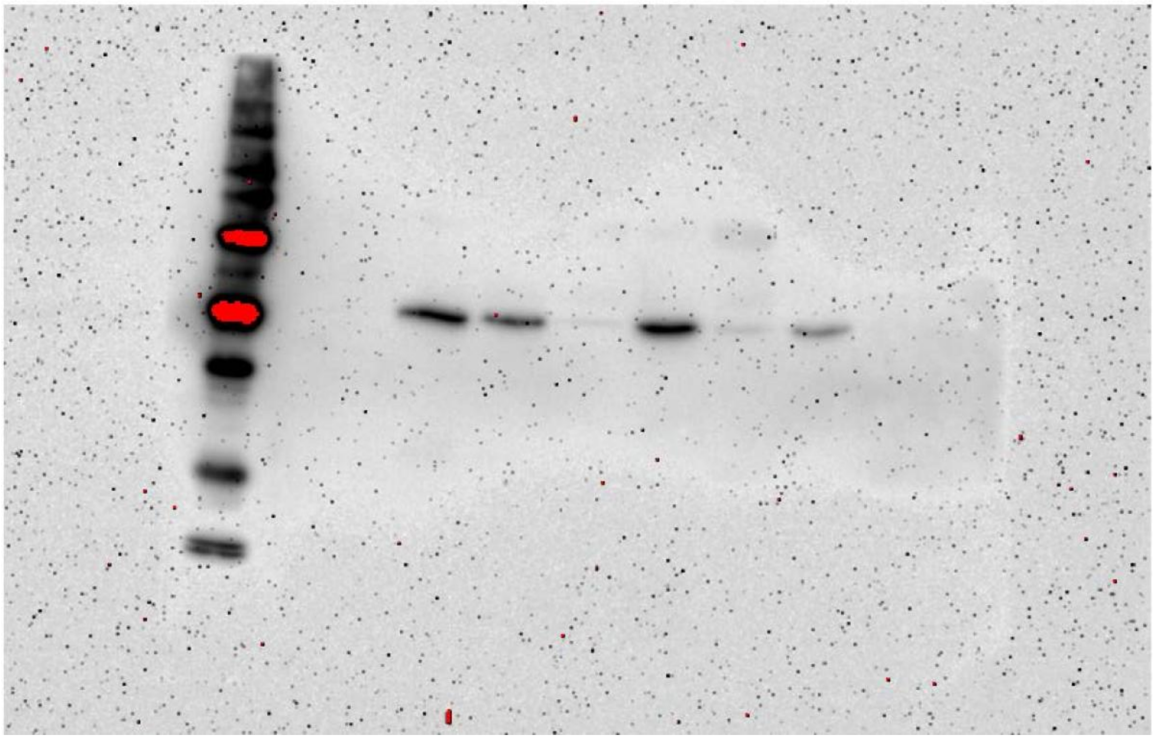

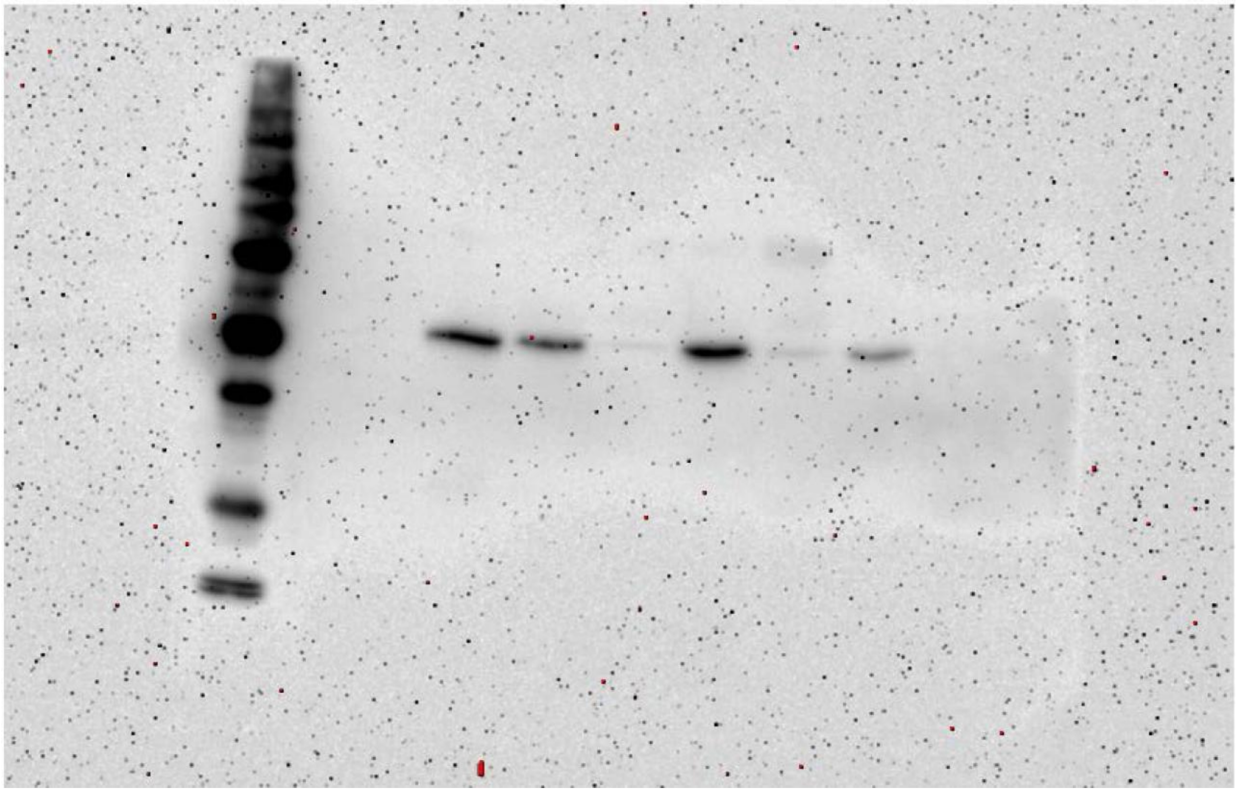

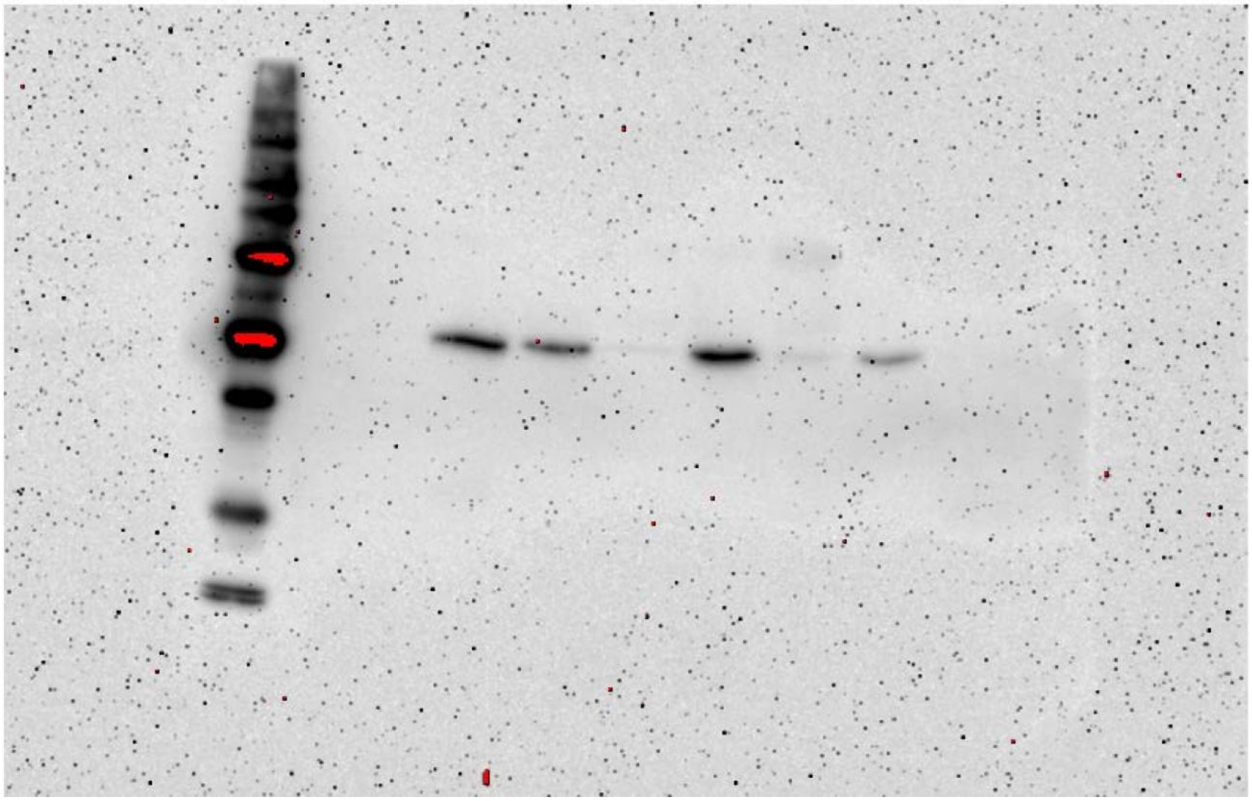

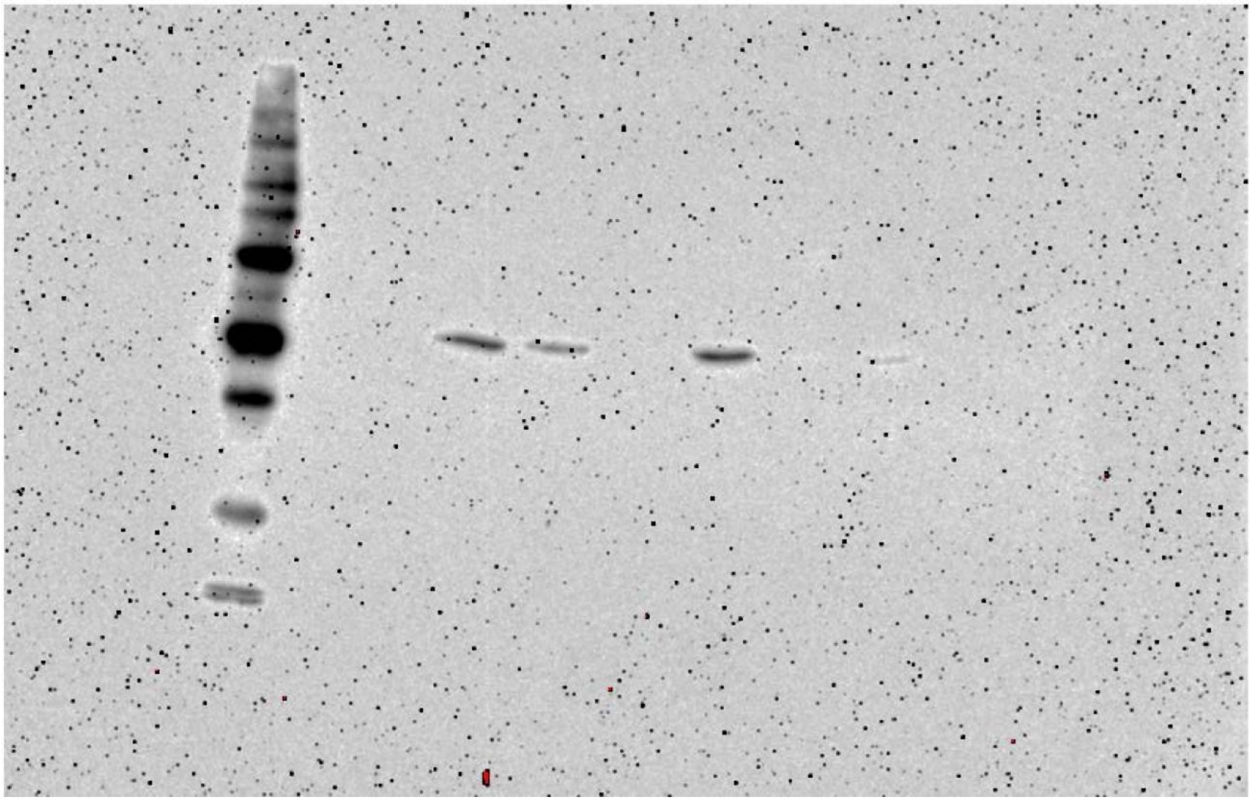

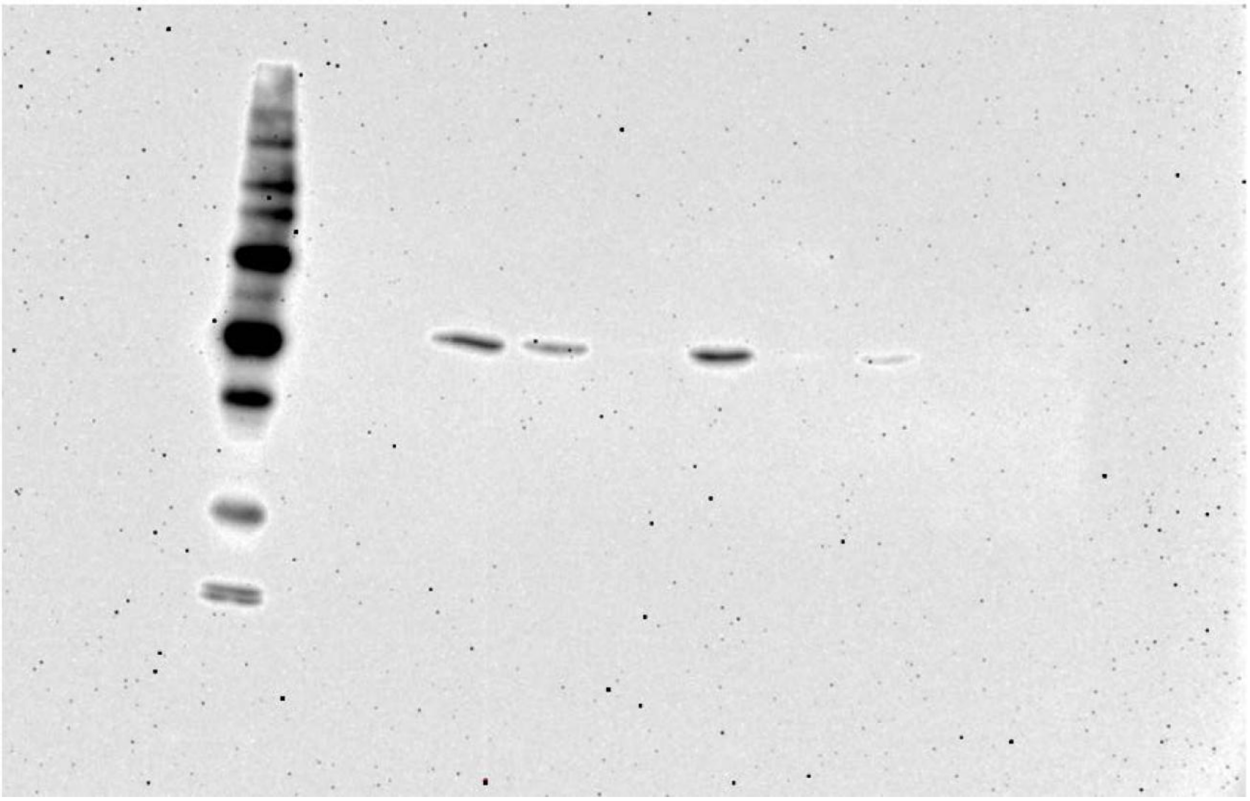

Supplement: S4 Fig — https://doi.org/10.6084/m9.figshare.28016408.v1. (PDF) [file pone.0313683.s004.pdf]
