# Supplementary material for: Untargeted lipidomics reveals unique lipid signatures of extracellular vesicles from porcine colostrum and milk
Source: PLoS One. 2025 Feb 13;20(2):e0313683. doi: 10.1371/journal.pone.0313683 (PMC11825007; doi:10.1371/journal.pone.0313683)
Supplement: S1 Table — https://doi.org/10.6084/m9.figshare.28016372.v2. (PDF) [file pone.0313683.s005.pdf]

S1 Table. Composition of basal sow diets and body condition score information

Composition of Basal Sow Diets.		
**Item**	**Gestation**	**Lactation**
Ingredients (g/kg as fed basis)		
Corn	284.60	249.10
Barley	224.20	216.70
Wheat bran	208.00	115.80
Distillers grains	125.00	40.00
Biscuit	50.20	52.10
Rice	35.00	35.00
Commercial concentrate *	25.00	250.00
Soybean oil	12.90	14.00
Fish meal	-	14.50
Mineral-vitamin premix **	20.00	11.70
HCl-Lysine	11.20	15.00
Composition (% DM)		
Crude protein	15.85	19.92
Crude fat	4.55	4.93
Crude fiber	5.69	5.66
Ash	5.68	4.46
Ca	1.70	1.21
P	0.56	0.57
Ca/P	3.04	2.12
Lysine	1.04	1.34
Methionine	0.18	0.22
Met +Cis	0.37	0.50

* Providing (as fed basis): 32.36% crude protein, 6.80% crude fat, 6.77% crude fiber, 0.80% Na, 2.43% lysine, 0.56% methionine. ** Providing (per kg of complete diet): vitamin A, 10,000 IU; vitamin D3, 2000 IU; vitamin E, 48 IU; vitamin K3, 1.5 mg; riboflavin, 6 mg; niacin, 40 mg; biotin, 0.2 mg; d-pantothenic, 17 mg; folic acid, 2 mg; choline, 166 mg; vitamin B6, 2 mg; and vitamin B12, 28 mg. Fe (as $FeSO_4$), 90 mg; Cu (as $CuSO_4$), 15 mg; Zn (as $ZnSO_4$), 50 mg; Mn (as MnO_2), 54 mg; I (as KI), 0.99 mg; and Se (as Na_2SeO_3), 0.25 mg.

Fatty Acid (g/100 g total fatty acids) of sow diets.				
**Item**	**Gestation CR**	**Gestation LR**	**Lactation CR**	**Lactation LR**
10:00	0.26	0.22	-	-
12:00	0.35	0.30	1.80	1.56
14:00	0.25	0.21	-	-
16:00	20.23	18.08	17.58	16.06
16:1 ω7	0.50	0.42	-	-
18:00	3.06	3.30	4.36	4.40
18:1 ω9 cis	19.88	19.80	24.51	23.84
18:1 ω7	1.05	1.03	-	0.12

18:2 ω6 cis 9,12	49.79	44.73	46.92	42.90
18:3 ω3	3.72	11.12	4.83	11.12
20:00	0.28	0.24	-	-
20:1 ω9	0.41	0.35	-	-
22:00	0.23	0.20	-	-
ω6	49.79	44.73	46.92	42.90
ω3	3.72	11.12	4.83	11.12
ω6:ω3	13.40	4.02	9.71	3.88

Control ratio (CR): sow diet with ω6:ω3 ratio = 13:1 during gestation, starting from day 28 (G28) and 10:1 during lactation; low ratio (LR): sow diet with ω6:ω3 ratio = 4:1 from G28 until the end of lactation.

	**Body Weight (kg, mean ± SD)**	**BCS (mean ± SD)**
Gestation Day 28	210 ± 25	2,44 ± 0,46
Gestation Day 79	242 ± 19	2,55 ± 0,53
Gestation Day 108	251 ± 19	2,88 ± 0,22
Lactation Day 26	217 ± 32	2,12 ± 0,64

The sow's body weight (BW) and body condition score (BCS) are presented by mean ± SD, and were calculated using all animals for proteomics experiment (n = 10). The BW and BCS were not statistically different between the feeding groups (p-value = 0.36 and 0.9, for BW and BCS respectively).
