# Supplementary material for: Untargeted lipidomics reveals unique lipid signatures of extracellular vesicles from porcine colostrum and milk
Source: PLoS One. 2025 Feb 13;20(2):e0313683. doi: 10.1371/journal.pone.0313683 (PMC11825007; doi:10.1371/journal.pone.0313683)
Supplement: S2 Table — https://doi.org/10.6084/m9.figshare.28016393.v1. (PDF) [file pone.0313683.s006.pdf]

S2 Table. List of analyzed lipids within their respective lipid species classes, subclasses and number of lipids annotated from the untargeted lipidomic analysis.

Categories	Main class	Lipid subclass	Lipid subclass abbreviation	Elements	% (Lipid elements in the respective category)
Fatty acyls [FA]	Oxygenated hydrocarbons	Oxidized fatty acids	OxFA	2	1,90
Fatty acyls [FA]	Fatty amides	N-acyl ornithine	NAOrn	3	2,86
Fatty acyls [FA]	Fatty amides	N-acyl glycyl serine	NAGlySer	4	3,81
Fatty acyls [FA]	Fatty amides	N-acyl glycine	NAGly	5	4,76
Fatty acyls [FA]	Fatty esters	Acylcarnitine	CAR	7	6,67
Fatty acyls [FA]	Fatty amides	N-acyl ethanolamines	NAE	34	32,38
Fatty acyls [FA]	Fatty acids and Conjugates	Free fatty acid	FA	50	47,62
Glycerolipids [GL]	Other Glycerolipids	Acyl diacylglyceryl glucuronide	ADGGA	1	0,26
Glycerolipids [GL]	Other Glycerolipids	ıethylhomoserine/diacylglyceryl hydroxymethyl-N,N,N-tı	DGTS	1	0,26
Glycerolipids [GL]	Other Glycerolipids	ıethylhomoserine/Lysodiacylglyceryl hydroxymethyl-N,N	LDGTS	1	0,26
Glycerolipids [GL]	Glycosyldiradylglycerols	Digalactosyldiacylglycerol	DGDG	3	0,77
Glycerolipids [GL]	Other Glycerolipids	Diacylglyceryl glucuronide	DGGA	3	0,77
Glycerolipids [GL]	Other Glycerolipids	Diacylglyceryl-3-O-carboxyhydroxymethylcholine	DGCC	4	1,02
Glycerolipids [GL]	Monoradylglycerols	Monoacylglycerol	MG	21	5,37
Glycerolipids [GL]	Triradylglycerols	Triacylglycerol	TG	172	43,99
Glycerolipids [GL]	Diradylglycerols	Diacylglycerol	DG	185	47,31
Glycerophospholipids [GP]	Glycerophosphates	Lysophosphatidic acid	LPA	1	0,46
Glycerophospholipids [GP]	Glycerophosphoethanolamines	N-monomethyl phosphatidylethanolamine	MMPE	1	0,46
Glycerophospholipids [GP]	Glycerophosphoserines	N-acyl-lysophosphatidylserine	LNAPS	1	0,46
Glycerophospholipids [GP]	Other Glycerophospholipids	Phosphatidylmethanol	PMeOH	1	0,46
Glycerophospholipids [GP]	Other Glycerophospholipids	Phosphatidylethanol	PEtOH	1	0,46
Glycerophospholipids [GP]	Glycerophosphoethanolamines	dimethyl-phosphatidylethanolamine	DMPE	2	0,93
Glycerophospholipids [GP]	Glycerophosphoglycerols	Bismonoacylglycerophosphate	BMP	3	1,39
Glycerophospholipids [GP]	Glycerophosphates	Phosphatidic acid	PA	5	2,31
Glycerophospholipids [GP]	Glycerophosphoglycerols	Hemibismonoacylglycerophosphate	HBMP	5	2,31
Glycerophospholipids [GP]	Glycerophosphocholines	Lysophophatidylcholine	LPC	6	2,78
Glycerophospholipids [GP]	Glycerophosphoglycerophosphoglycerols	Cardiolipin	CL	8	3,70
Glycerophospholipids [GP]	Glycerophosphoethanolamines	Lysophosphatidylethanolamine	LPE	9	4,17
Glycerophospholipids [GP]	Glycerophosphoglycerols	Phosphatidylglycerol	PG	9	4,17
Glycerophospholipids [GP]	Glycerophosphoserines	Phosphatidylserine	PS	14	6,48
Glycerophospholipids [GP]	Glycerophosphoinositols	Phosphatidylinositol	PI	24	11,11
Glycerophospholipids [GP]	Glycerophosphoethanolamines	Phosphatidylethanolamine	PE	59	27,31
Glycerophospholipids [GP]	Glycerophosphocholines	Phosphatidylcholine	PC	67	31,02
Prenol Lipids [PR]	Quinones and hydroquinones	Coenzyme Q	CoQ10	1	100
Sphingolipids [SP]	Phosphosphingolipids	Ceramide phosphoethanolamine	PE-Cer	4	2,11
Sphingolipids [SP]	Phosphosphingolipids	Ceramide phosphoinositol	PI-Cer	5	2,63
Sphingolipids [SP]	Neutral glycosphingolipids	Acylhexosylceramide	AHexCer	7	3,68
Sphingolipids [SP]	Ceramides	Ceramide 1-phosphates	CerP	8	4,21
Sphingolipids [SP]	Acidic glycosphingolipids	Sulfatide	SHexCer	10	5,26
Sphingolipids [SP]	Neutral glycosphingolipids	Hexosylceramide	HexCer	12	6,32
Sphingolipids [SP]	Sphingoid bases	Sulfonolipid	SL	14	7,37
Sphingolipids [SP]	Ceramides	Ceramides	Cer	61	32,11
Sphingolipids [SP]	Phosphosphingolipids	Sphingomyelin	SM	69	36,32
Sterol Lipids [ST]	Secosteroids	Vitamin D	25-hydroxycholecalciferol	1	2,27
Sterol Lipids [ST]	Sterols	Cholesteryl ester	CE	1	2,27
Sterol Lipids [ST]	Sterols	Sterols	ST	18	40,91
Sterol Lipids [ST]	Sterols	Sterol esters	SE	24	54,55
