# Supplementary material for: Untargeted lipidomics reveals unique lipid signatures of extracellular vesicles from porcine colostrum and milk
Source: PLoS One. 2025 Feb 13;20(2):e0313683. doi: 10.1371/journal.pone.0313683 (PMC11825007; doi:10.1371/journal.pone.0313683)
Supplement: S3 Table — https://doi.org/10.6084/m9.figshare.28016402.v1. (PDF) [file pone.0313683.s007.pdf]

S3 Table. List of all significantly up- and down-regulated lipids elements in comparison between porcine colostrum exosomes (day 0) and milk exosomes at day 7. Significance was set using t-test FDR adjusted p-value threshold at 0.05 and fold change threshold at 2 ($|\log_2 FC > 1|$).

**Day 0 vs 7**				
**Lipid class**	**Lipid name**	**log2(FC)**	**FDR Ajusted P value**	**Up/Down**
ADGGA	ADGGA (O-28:0)17:2_22:6	1.253	0.007	UP
AHexCer	AHexCer 59:9;3O	-3.162	0.000	Down
AHexCer	AHexCer 40:4;3O	-2.493	0.000	Down
AHexCer	AHexCer 39:4;3O_AHexCer (O-14:1)25:3;3O	-2.029	0.000	Down
AHexCer	AHexCer 60:8;3O	-1.653	0.001	Down
AHexCer	AHexCer 72:9;3O	-1.451	0.002	Down
BMP	BMP 17:1_17:1	-3.217	0.000	Down
BMP	BMP 15:0_8:0	-2.698	0.000	Down
BMP	BMP 8:0_28:2	-2.083	0.000	Down
CAR	CAR 17:3	-3.046	0.000	Down
CAR	CAR 13:0	-2.731	0.000	Down
CAR	CAR 7:0	-2.701	0.000	Down
CAR	CAR 5:0	-2.655	0.000	Down
CAR	CAR 19:3	-2.167	0.000	Down
CAR	CAR 16:0	-1.693	0.000	Down
CE	CE 18:1	-2.549	0.000	Down
Cer	Cer 34:1;2O_Cer 18:1;2O_16:0,	1.028	0.005	UP
Cer	Cer 12:0;2O_19:0;(2OH)	1.807	0.000	UP
Cer	Cer 12:1;2O_24:3;(2OH)	3.046	0.000	UP
Cer	Cer 13:2;2O_40:3	-4.407	0.000	Down
Cer	Cer 65:5;4O	-3.724	0.000	Down
Cer	Cer 38:0;2O_Cer 18:0;2O_20:0	-3.406	0.000	Down
Cer	Cer 40:7;4O	-3.382	0.000	Down
Cer	Cer 12:0;2O_23:0;O	-3.238	0.000	Down
Cer	Cer 42:0;4O	-3.126	0.000	Down
Cer	Cer 33:6;4O	-3.101	0.000	Down
Cer	Cer 50:10;4O	-3.024	0.000	Down
Cer	Cer 40:0;4O_Cer 30:0;3O_10:0;(2OH)	-3.009	0.000	Down
Cer	Cer 34:3;4O_Cer 19:2;3O_15:1;(2OH)	-2.952	0.000	Down
Cer	Cer 24:3;3O_Cer 16:3;2O_8:0;O	-2.946	0.000	Down
Cer	Cer 38:3;5O_Cer 21:2;3O_17:1;(2OH)	-2.909	0.000	Down
Cer	Cer 35:5;5O_Cer 19:2;3O_16:3;(2OH)	-2.904	0.000	Down
Cer	Cer 36:4;4O	-2.896	0.000	Down
Cer	Cer 36:1;3O_Cer 19:0;2O_17:1;O	-2.842	0.000	Down
Cer	Cer 36:2;3O_Cer 19:0;2O_17:2;O	-2.830	0.000	Down
Cer	Cer 38:5;4O	-2.786	0.000	Down
Cer	Cer 33:5;4O	-2.754	0.000	Down
Cer	Cer 34:0;3O_Cer 18:0;2O_16:0;O	-2.749	0.000	Down
Cer	Cer 34:1;3O_Cer 19:0;2O_15:1;O	-2.733	0.000	Down
Cer	Cer 35:5;4O	-2.707	0.000	Down

Cer	Cer 28:3;4O,	-2.690	0.000	Down
Cer	Cer 28:3;4O	-2.630	0.000	Down
Cer	Cer 35:6;2O_Cer 17:3;2O_18:3	-2.588	0.000	Down
Cer	Cer 94:3;4O	-2.443	0.000	Down
Cer	Cer 60:11;4O	-2.288	0.000	Down
Cer	Cer 58:11;4O	-2.282	0.000	Down
Cer	Cer 12:2;2O_24:1	-2.274	0.000	Down
Cer	Cer 12:1;3O_31:0;(2OH)	-2.272	0.000	Down
Cer	Cer 12:2;2O_39:10;2O	-2.255	0.000	Down
Cer	Cer 12:1;2O_30:0	-2.201	0.000	Down
Cer	Cer 42:1;2O_Cer 18:1;2O_24:0,	-2.061	0.000	Down
Cer	Cer 92:5;4O	-2.013	0.000	Down
Cer	Cer 42:1;2O_Cer 18:1;2O_24:0	-1.991	0.000	Down
Cer	Cer 31:4;2O_Cer 12:2;2O_19:2	-1.799	0.000	Down
Cer	Cer 58:5;4O	-1.688	0.001	Down
Cer	Cer 94:5;4O	-1.652	0.001	Down
Cer	Cer 61:13;4O	-1.627	0.001	Down
Cer	Cer 12:2;2O_42:10;2O	-1.617	0.001	Down
Cer	Cer 16:3;2O_30:3	-1.551	0.000	Down
Cer	Cer 12:2;2O_30:0	-1.359	0.000	Down
Cer	Cer 42:2;2O_Cer 18:1;2O_24:1	-1.334	0.000	Down
Cer	Cer 12:1;2O_28:0	-1.192	0.000	Down
Cer	Cer 42:1;3O_Cer 18:1;2O_24:0;O	-1.097	0.000	Down
CerP	CerP 24:2;2O_28:3	-3.030	0.000	Down
CerP	CerP 29:2;2O_CerP 16:1;2O_13:1	-2.605	0.000	Down
CerP	CerP 30:3;2O_CerP 12:0;2O_18:3	-2.356	0.000	Down
CerP	CerP 34:2;2O_CerP 19:1;2O_15:1	-1.854	0.000	Down
CerP	CerP 16:1;2O_28:3	-1.473	0.001	Down
CerP	CerP 14:1;2O_28:3	-1.082	0.006	Down
CL	CL 14:1_22:6_26:0_28:0	-3.012	0.000	Down
CL	CL 12:0_22:6_28:0_28:0	-2.901	0.000	Down
CL	CL 72:6_CL 18:0_18:0_16:1_20:5	-2.126	0.000	Down
CL	CL 15:0_22:5_28:0_28:0	-1.626	0.002	Down
CoQ10	CoQ10	-2.013	0.000	Down
DG	DG 47:6	-4.354	0.000	Down
DG	DG 42:0	-4.345	0.000	Down
DG	DG 86:5	-4.335	0.000	Down
DG	DG 43:2	-4.300	0.000	Down
DG	DG 50:6	-4.293	0.000	Down
DG	DG 41:0	-4.291	0.000	Down
DG	DG 43:0	-4.213	0.000	Down
DG	DG 51:4	-4.143	0.000	Down
DG	DG 28:0	-4.090	0.000	Down
DG	DG O-45:6_DG O-17:0_28:6	-4.072	0.000	Down
DG	DG 39:1	-4.020	0.000	Down
DG	DG 44:0	-3.991	0.000	Down
DG	DG 42:5	-3.980	0.000	Down
DG	DG 40:0	-3.979	0.000	Down

DG	DG 45:0	-3.950	0.000	Down
DG	DG 52:6	-3.950	0.000	Down
DG	DG 46:0	-3.915	0.000	Down
DG	DG 48:6	-3.903	0.000	Down
DG	DG 49:6	-3.899	0.000	Down
DG	DG 49:7	-3.892	0.000	Down
DG	DG O-42:6_DG O-18:0_24:6	-3.890	0.000	Down
DG	DG 45:6	-3.889	0.000	Down
DG	DG 46:6	-3.853	0.000	Down
DG	DG 30:0	-3.823	0.000	Down
DG	DG 39:6	-3.822	0.000	Down
DG	DG 51:0	-3.797	0.000	Down
DG	DG 49:8	-3.796	0.000	Down
DG	DG 47:0	-3.793	0.000	Down
DG	DG 50:0	-3.789	0.000	Down
DG	DG 43:6	-3.789	0.000	Down
DG	DG 49:0	-3.783	0.000	Down
DG	DG 38:6	-3.781	0.000	Down
DG	DG 39:0	-3.770	0.000	Down
DG	DG 45:7	-3.765	0.000	Down
DG	DG O-55:1_DG O-27:0_28:1	-3.758	0.000	Down
DG	DG O-38:1_DG O-14:0_24:1	-3.756	0.000	Down
DG	DG 40:5	-3.752	0.000	Down
DG	DG O-41:1_DG O-13:0_28:1	-3.742	0.000	Down
DG	DG O-48:1_DG O-20:0_28:1	-3.734	0.000	Down
DG	DG 35:6	-3.728	0.000	Down
DG	DG 35:0	-3.725	0.000	Down
DG	DG O-39:1_DG O-13:0_26:1	-3.716	0.000	Down
DG	DG 43:7	-3.710	0.000	Down
DG	DG 40:2	-3.689	0.000	Down
DG	DG 41:7	-3.680	0.000	Down
DG	DG 39:7	-3.673	0.000	Down
DG	DG 41:6	-3.654	0.000	Down
DG	DG O-44:1_DG O-18:0_26:1	-3.645	0.000	Down
DG	DG O-40:1_DG O-16:0_24:1	-3.641	0.000	Down
DG	DG 36:0	-3.631	0.000	Down
DG	DG 27:0	-3.627	0.000	Down
DG	DG 37:7	-3.617	0.000	Down
DG	DG 51:6	-3.609	0.000	Down
DG	DG 34:0	-3.544	0.000	Down
DG	DG 36:0_DG 18:0_18:0	-3.531	0.000	Down
DG	DG 40:6	-3.514	0.000	Down
DG	DG 45:8	-3.513	0.000	Down
DG	DG 43:8	-3.508	0.000	Down
DG	DG 38:2	-3.502	0.000	Down
DG	DG 44:6_DG 16:0_28:6	-3.493	0.000	Down
DG	DG O-35:0_DG O-19:0_16:0	-3.483	0.000	Down
DG	DG 52:7	-3.483	0.000	Down

DG	DG 42:6_DG 16:0_26:6	-3.478	0.000	Down
DG	DG O-30:1_DG O-16:0_14:1	-3.451	0.000	Down
DG	DG 32:0	-3.437	0.000	Down
DG	DG 31:0	-3.405	0.000	Down
DG	DG 27:4	-3.400	0.000	Down
DG	DG 36:6	-3.397	0.000	Down
DG	DG O-36:1_DG O-17:0_19:1	-3.395	0.000	Down
DG	DG 33:1	-3.387	0.000	Down
DG	DG 34:2_DG 16:0_18:2	-3.373	0.000	Down
DG	DG O-33:1_DG O-17:0_16:1	-3.360	0.000	Down
DG	DG 48:12	-3.327	0.000	Down
DG	DG 52:11	-3.301	0.000	Down
DG	DG 34:0_DG 16:0_18:0	-3.265	0.000	Down
DG	DG 27:5	-3.259	0.000	Down
DG	DG 52:14	-3.259	0.000	Down
DG	DG 42:10	-3.235	0.000	Down
DG	DG 32:0_DG 16:0_16:0	-3.190	0.000	Down
DG	DG 40:9	-3.176	0.000	Down
DG	DG 41:6_DG 15:0_26:6	-3.136	0.000	Down
DG	DG 31:4	-3.132	0.000	Down
DG	DG 51:7	-3.125	0.000	Down
DG	DG 46:3	-3.108	0.000	Down
DG	DG 30:5	-3.099	0.000	Down
DG	DG 38:5	-3.099	0.000	Down
DG	DG 38:8	-3.088	0.000	Down
DG	DG 30:7	-3.085	0.000	Down
DG	DG 16:0	-3.078	0.000	Down
DG	DG 45:11	-3.073	0.000	Down
DG	DG 35:2	-3.067	0.000	Down
DG	DG 42:11	-3.050	0.000	Down
DG	DG 30:8	-3.028	0.000	Down
DG	DG 32:6	-3.017	0.000	Down
DG	DG 41:11	-3.010	0.000	Down
DG	DG 28:2	-3.004	0.000	Down
DG	DG 49:2	-3.003	0.000	Down
DG	DG 27:3	-3.000	0.000	Down
DG	DG 38:7	-2.997	0.000	Down
DG	DG 39:10	-2.996	0.000	Down
DG	DG 19:0	-2.995	0.000	Down
DG	DG 30:4	-2.995	0.000	Down
DG	DG 24:1	-2.965	0.000	Down
DG	DG 34:5	-2.958	0.000	Down
DG	DG 24:2	-2.953	0.000	Down
DG	DG 46:11	-2.946	0.000	Down
DG	DG 36:5	-2.937	0.000	Down
DG	DG 32:2	-2.934	0.000	Down
DG	DG 33:7	-2.931	0.000	Down
DG	DG 37:4	-2.919	0.000	Down

DG	DG 34:3	-2.915	0.000	Down
DG	DG 26:0	-2.905	0.000	Down
DG	DG 32:7	-2.905	0.000	Down
DG	DG 44:6	-2.904	0.000	Down
DG	DG 28:4	-2.904	0.000	Down
DG	DG 29:3	-2.896	0.000	Down
DG	DG 29:4	-2.895	0.000	Down
DG	DG 36:4	-2.893	0.000	Down
DG	DG 32:8	-2.889	0.000	Down
DG	DG 26:2	-2.889	0.000	Down
DG	DG 30:6	-2.872	0.000	Down
DG	DG 29:5	-2.869	0.000	Down
DG	DG 20:0	-2.862	0.000	Down
DG	DG 31:7	-2.862	0.000	Down
DG	DG 32:3	-2.858	0.000	Down
DG	DG 25:2	-2.856	0.000	Down
DG	DG 30:3	-2.848	0.000	Down
DG	DG 26:5	-2.848	0.000	Down
DG	DG 29:0	-2.845	0.000	Down
DG	DG 31:8	-2.845	0.000	Down
DG	DG 23:0	-2.845	0.000	Down
DG	DG 31:5	-2.845	0.000	Down
DG	DG 32:1	-2.842	0.000	Down
DG	DG 25:0	-2.832	0.000	Down
DG	DG 25:1	-2.830	0.000	Down
DG	DG 28:3	-2.829	0.000	Down
DG	DG 23:4	-2.819	0.000	Down
DG	DG 39:9	-2.817	0.000	Down
DG	DG 23:1	-2.815	0.000	Down
DG	DG 28:5	-2.814	0.000	Down
DG	DG 40:8	-2.812	0.000	Down
DG	DG 39:8	-2.795	0.000	Down
DG	DG 23:2	-2.785	0.000	Down
DG	DG 31:6	-2.775	0.000	Down
DG	DG 24:0	-2.772	0.000	Down
DG	DG 36:1_DG 18:0_18:1	-2.771	0.000	Down
DG	DG 34:4	-2.770	0.000	Down
DG	DG 27:2	-2.765	0.000	Down
DG	DG 34:2	-2.744	0.000	Down
DG	DG 22:0	-2.681	0.000	Down
DG	DG 29:2	-2.678	0.000	Down
DG	DG 18:0	-2.657	0.000	Down
DG	DG 24:3	-2.616	0.000	Down
DG	DG 51:8	-2.607	0.000	Down
DG	DG 22:1	-2.566	0.000	Down
DG	DG 30:2	-2.464	0.000	Down
DG	DG 46:8	-2.451	0.000	Down
DG	DG 36:1	-2.361	0.000	Down

DG	DG 44:7	-2.252	0.000	Down
DG	DG 34:1_DG 16:0_18:1	-2.243	0.000	Down
DG	DG 36:2	-2.223	0.000	Down
DG	DG 34:1	-2.107	0.000	Down
DG	DG 51:13	-2.067	0.000	Down
DG	DG 52:10	-1.945	0.001	Down
DG	DG 48:13	-1.935	0.000	Down
DG	DG 36:2_DG 18:1_18:1	-1.899	0.000	Down
DG	DG 52:9	-1.860	0.000	Down
DG	DG 28:1	-1.840	0.000	Down
DG	DG 36:3_DG 18:1_18:2	-1.783	0.000	Down
DG	DG 41:8	-1.578	0.000	Down
DG	DG 50:1	-1.574	0.000	Down
DG	DG 38:4	-1.542	0.001	Down
DG	DG 50:9	-1.363	0.004	Down
DG	DG 53:9	-1.185	0.003	Down
DG	DG 49:1	-1.028	0.016	Down
DGCC	DGCC 36:2_DGCC 18:1_18:1	-3.236	0.000	Down
DGCC	DGCC 16:0_19:5	-1.752	0.000	Down
DGCC	DGCC 15:2_18:5	-1.695	0.000	Down
DGCC	DGCC 15:0_22:6	-1.583	0.000	Down
DGDG	DGDG 8:0_20:2	-1.997	0.000	Down
DGDG	DGDG O-8:0_17:0	-1.714	0.000	Down
DGDG	DGDG 17:1_22:6	-1.549	0.000	Down
DGGA	DGGA 10:0_22:1	-2.713	0.000	Down
DGGA	DGGA 22:0_22:6	-1.995	0.000	Down
DGGA	DGGA 12:0_22:1	-1.659	0.000	Down
DGTS	DGTS 16:0_17:3	-3.016	0.000	Down
DMPE	DMPE 17:0_22:5	-1.816	0.000	Down
FA	FA 20:4;3O	-3.930	0.000	Down
FA	FA 16:0;3O	-3.859	0.000	Down
FA	FA 16:1;3O	-3.689	0.000	Down
FA	FA 44:5	-3.651	0.000	Down
FA	FA 40:5	-3.592	0.000	Down
FA	FA 42:5	-3.586	0.000	Down
FA	FA 16:1	-3.493	0.000	Down
FA	FA 15:4	-3.486	0.000	Down
FA	FA 42:9	-3.472	0.000	Down
FA	FA 18:3;4O	-3.355	0.000	Down
FA	FA 16:2;3O	-3.313	0.000	Down
FA	FA 22:0;4O	-3.180	0.000	Down
FA	FA 19:4;1O	-3.073	0.000	Down
FA	FA 17:4;2O	-2.955	0.000	Down
FA	FA 38:5	-2.914	0.000	Down
FA	FA 19:1;2O	-2.864	0.000	Down
FA	FA 20:0;4O	-2.806	0.000	Down
FA	FA 20:3;4O	-2.804	0.000	Down
FA	FA 22:6	-2.775	0.000	Down

FA	FA 25:0	-2.715	0.000	Down
FA	FA 22:5;4O	-2.664	0.000	Down
FA	FA 14:0	-2.610	0.000	Down
FA	FA 28:7	-2.608	0.000	Down
FA	FA 22:6;4O	-2.581	0.000	Down
FA	FA 29:0	-2.557	0.000	Down
FA	FA 32:0	-2.534	0.000	Down
FA	FA 30:0	-2.525	0.000	Down
FA	FA 26:0	-2.516	0.000	Down
FA	FA 24:0	-2.504	0.000	Down
FA	FA 31:0	-2.493	0.000	Down
FA	FA 27:0	-2.449	0.000	Down
FA	FA 18:1	-2.440	0.000	Down
FA	FA 20:0	-2.413	0.000	Down
FA	FA 23:0	-2.412	0.000	Down
FA	FA 16:0	-2.408	0.000	Down
FA	FA 28:1;2O	-2.399	0.000	Down
FA	FA 28:0	-2.364	0.000	Down
FA	FA 34:0	-2.362	0.000	Down
FA	FA 18:0	-2.346	0.000	Down
FA	FA 22:0	-2.341	0.000	Down
FA	FA 17:0	-2.325	0.000	Down
FA	FA 26:1;1O	-2.280	0.000	Down
FA	FA 36:5	-2.217	0.000	Down
FA	FA 33:0	-2.169	0.000	Down
FA	FA 18:1;O	-2.084	0.000	Down
FA	FA 15:0	-1.822	0.000	Down
FA	FA 18:1;2O	-1.734	0.000	Down
FA	FA 21:0	-1.724	0.005	Down
FA	FA 42:10	1.747	0.000	UP
FA	FA 44:10	2.220	0.000	UP
HBMP	HBMP 22:2_12:0_12:0	-3.143	0.000	Down
HBMP	HBMP 20:1_12:0_12:0	-2.698	0.000	Down
HBMP	HBMP 22:3_12:0_12:0	-2.210	0.000	Down
HBMP	HBMP 13:1_12:0_13:1	-1.094	0.001	Down
HexCer	HexCer 18:1;2O_18:5	-3.642	0.000	Down
HexCer	HexCer 16:1;3O_17:0;(2OH)	-3.555	0.000	Down
HexCer	HexCer 16:0;2O_24:1	-3.203	0.000	Down
HexCer	HexCer 16:1;3O_26:7;(2OH)	-2.961	0.000	Down
HexCer	HexCer 18:0;2O_18:5	-2.109	0.000	Down
HexCer	HexCer 16:0;2O_30:4;O	-1.874	0.000	Down
HexCer	HexCer 34:0;2O	-1.557	0.000	Down
HexCer	HexCer 34:1;3O_HexCer 18:1;2O_16:0;O	-1.502	0.000	Down
HexCer	HexCer 20:2;2O_20:5	-1.335	0.010	Down
LDGTS	LDGTS 15:0	-2.660	0.000	Down
LNAPS	LNAPS 14:0_N-28:0	-2.309	0.000	Down
LPA	LPA 28:2	-3.049	0.000	Down
LPC	LPC 28:7	-2.267	0.000	Down

LPC	LPC 38:6	-1.310	0.007	Down
LPC	LPC 38:5	-1.301	0.002	Down
LPC	LPC 18:0	1.306	0.004	UP
LPE	LPE O-17:1	-2.884	0.000	Down
LPE	LPE 18:1,	-2.055	0.000	Down
LPE	LPE 16:0	-1.784	0.001	Down
LPE	LPE 18:1	-1.672	0.000	Down
LPE	LPE O-16:1,	1.950	0.003	UP
LPE	LPE O-18:1	2.851	0.000	UP
MG	MG 22:5	-3.705	0.000	Down
MG	MG 12:0	-3.486	0.000	Down
MG	MG 17:0	-2.962	0.000	Down
MG	MG 16:0	-2.914	0.000	Down
MG	MG 18:0	-2.914	0.000	Down
MG	MG 15:4	-2.880	0.000	Down
MG	MG 18:3	-2.844	0.000	Down
MG	MG 15:0	-2.843	0.000	Down
MG	MG 17:4	-2.838	0.000	Down
MG	MG 15:3	-2.806	0.000	Down
MG	MG 15:2	-2.800	0.000	Down
MG	MG 19:5	-2.775	0.000	Down
MG	MG 10:0	-2.731	0.000	Down
MG	MG 16:3	-2.729	0.000	Down
MG	MG 9:0	-2.689	0.000	Down
MG	MG 21:1	-2.579	0.000	Down
MG	MG 13:0	-2.563	0.000	Down
MG	MGDG O-16:4_22:6	-2.267	0.000	Down
NAE	NAE 16:1	-3.394	0.000	Down
NAE	NAE 26:5	-3.287	0.000	Down
NAE	NAE 19:5	-3.237	0.000	Down
NAE	NAE 26:6	-3.179	0.000	Down
NAE	NAE 17:4	-3.126	0.000	Down
NAE	NAE 24:5	-3.117	0.000	Down
NAE	NAE 14:1	-3.103	0.000	Down
NAE	NAE 18:4	-3.102	0.000	Down
NAE	NAE 14:0	-3.100	0.000	Down
NAE	NAE 20:3	-3.087	0.000	Down
NAE	NAE 15:1	-3.018	0.000	Down
NAE	NAE 18:3	-2.995	0.000	Down
NAE	NAE 22:5	-2.981	0.000	Down
NAE	NAE 20:5	-2.980	0.000	Down
NAE	NAE 18:5	-2.936	0.000	Down
NAE	NAE 18:1	-2.931	0.000	Down
NAE	NAE 18:2	-2.929	0.000	Down
NAE	NAE 16:0	-2.908	0.000	Down
NAE	NAE 15:4	-2.901	0.000	Down
NAE	NAE 20:4	-2.900	0.000	Down
NAE	NAE 20:2	-2.900	0.000	Down

NAE	NAE 16:4	-2.899	0.000	Down
NAE	NAE 13:1	-2.888	0.000	Down
NAE	NAE 21:4	-2.881	0.000	Down
NAE	NAE 16:3	-2.877	0.000	Down
NAE	NAE 19:4	-2.873	0.000	Down
NAE	NAE 6:0	-2.837	0.000	Down
NAE	NAE 15:0	-2.814	0.000	Down
NAE	NAE 22:4	-2.770	0.000	Down
NAE	NAE 22:3	-2.767	0.000	Down
NAE	NAE 15:3	-2.746	0.000	Down
NAE	NAE 20:1	-2.693	0.000	Down
NAE	NAE 7:0	-2.685	0.000	Down
NAE	NAE 16:2	-2.255	0.000	Down
NAGly	NAGly 22:6_21:5	-3.689	0.000	Down
NAGly	NAGly 17:0;O	-2.884	0.000	Down
NAGly	NAGly 13:1;O	-2.878	0.000	Down
NAGly	NAGly 21:1_9:0	-2.540	0.000	Down
NAGlySer	NAGly 30:0_NAGly 20:0_10:0	-2.971	0.000	Down
NAGlySer	NAGlySer 22:6_20:1	-1.992	0.000	Down
NAOrn	NAOrn 13:0;O	-2.830	0.000	Down
NAOrn	NAOrn 14:1;O	-2.770	0.000	Down
NAOrn	NAGlySer 22:6_21:4	-1.507	0.003	Down
OxFA	OxFA 18:0;(2OH)	-2.866	0.000	Down
OxFA	NAOrn 22:2_20:0	-1.715	0.000	Down
PA	PA 23:0_28:7	-1.817	0.000	Down
PA	OxFA 18:2;(2OH)	-1.511	0.000	Down
PA	PA 17:0_28:6	-1.330	0.004	Down
PA	PA 15:0_28:7	-1.171	0.014	Down
PA	PA 21:0_28:6	-1.055	0.020	Down
PC	PC O-39:0	-4.053	0.000	Down
PC	PC 30:1	-3.735	0.000	Down
PC	PC O-30:0	-3.540	0.000	Down
PC	PC O-32:1	-3.362	0.000	Down
PC	PC O-12:0_22:5;4O	-3.251	0.000	Down
PC	PC 32:2_PC 16:1_16:1	-3.241	0.000	Down
PC	PC 32:1_PC 16:0_16:1	-3.213	0.000	Down
PC	PC 30:0	-3.200	0.000	Down
PC	PC 15:0_18:1(d7)	-3.127	0.000	Down
PC	PC O-35:7	-3.117	0.000	Down
PC	PC 28:0_PC 12:0_16:0	-3.079	0.000	Down
PC	PC O-32:0	-2.978	0.000	Down
PC	PC O-10:0_22:3;4O	-2.885	0.000	Down
PC	PC 32:1	-2.756	0.000	Down
PC	PC 34:3_PC 16:1_18:2	-2.702	0.000	Down
PC	PC O-36:7	-2.657	0.000	Down
PC	PC 33:1	-2.388	0.000	Down
PC	PC O-39:3	-2.364	0.000	Down
PC	PC 32:0,	-2.309	0.000	Down

PC	PC O-34:0	-2.269	0.000	Down
PC	PC 34:2	-2.221	0.000	Down
PC	PC O-34:1	-2.198	0.000	Down
PC	PC O-37:1	-2.192	0.000	Down
PC	PC O-37:8	-2.177	0.000	Down
PC	PC 36:4_PC 18:2_18:2	-2.125	0.001	Down
PC	PC 34:3	-2.098	0.001	Down
PC	PC O-14:1_24:0;1O	-2.097	0.000	Down
PC	PC 34:2_PC 16:0_18:2	-1.981	0.001	Down
PC	PC 34:1_PC 16:0_18:1	-1.944	0.000	Down
PC	PC O-14:0_22:5;3O	-1.912	0.000	Down
PC	PC 33:0	-1.900	0.000	Down
PC	PC 32:0_PC 16:0_16:0	-1.891	0.000	Down
PC	PC O-18:0_18:1;1O	-1.883	0.000	Down
PC	PC O-38:7	-1.858	0.000	Down
PC	PC O-36:4	-1.770	0.000	Down
PC	PC 36:0	-1.758	0.000	Down
PC	PC 38:6	-1.719	0.000	Down
PC	PC 36:3	-1.682	0.001	Down
PC	PC 32:0	-1.636	0.000	Down
PC	PC 34:0_PC 16:0_18:0	-1.617	0.000	Down
PC	PC 36:1	-1.604	0.000	Down
PC	PC 34:1	-1.591	0.000	Down
PC	PC O-12:0_22:3;2O	-1.560	0.001	Down
PC	PC 10:0_26:1	-1.546	0.001	Down
PC	PC 40:5	-1.522	0.004	Down
PC	PC 35:1	-1.501	0.000	Down
PC	PC O-39:10	-1.487	0.000	Down
PC	PC 37:6,	-1.475	0.001	Down
PC	PC 35:2	-1.457	0.001	Down
PC	PC O-39:8	-1.456	0.000	Down
PC	PC 39:2	-1.431	0.003	Down
PC	PC O-30:7	-1.398	0.000	Down
PC	PC O-37:9	-1.332	0.001	Down
PC	PC O-39:7	-1.328	0.001	Down
PC	PC 37:6	-1.304	0.009	Down
PC	PC 38:5	-1.285	0.008	Down
PC	PC 36:1_PC 18:0_18:1	-1.282	0.002	Down
PC	PC 36:2	-1.260	0.009	Down
PC	PC 36:3_PC 18:1_18:2	-1.133	0.016	Down
PC	PC 39:8	-1.057	0.005	Down
PC	PC 40:6	-1.043	0.001	Down
PC	PC 36:2_PC 18:0_18:2	-1.023	0.038	Down
PE	PE 32:1_PE 16:0_16:1	-4.760	0.000	Down
PE	PE 34:3	-4.371	0.000	Down
PE	PE O-32:2_PE O-16:1_16:1	-4.067	0.000	Down
PE	PE 32:1	-3.831	0.000	Down
PE	PE O-26:6_14:0	-3.618	0.000	Down

PE	PE P-32:1_PE P-16:0_16:1	-3.463	0.000	Down
PE	PE P-32:0_PE P-16:0_16:0	-3.334	0.000	Down
PE	PE O-32:1_PE O-16:1_16:0	-3.317	0.000	Down
PE	PE 32:0_PE 16:0_16:0	-3.297	0.000	Down
PE	PE 34:2_PE 16:1_18:1	-3.118	0.000	Down
PE	PE 34:2_PE 16:0_18:2	-2.991	0.000	Down
PE	PE 34:1_PE 16:0_18:1	-2.927	0.000	Down
PE	PE P-34:1_PE P-16:0_18:1	-2.915	0.000	Down
PE	PE 34:1_PE 16:0_18:1,	-2.874	0.000	Down
PE	PE 34:2	-2.827	0.000	Down
PE	PE O-34:2_PE O-16:1_18:1	-2.801	0.000	Down
PE	PE P-36:2_PE P-18:1_18:1	-2.764	0.000	Down
PE	PE O-18:3_22:5;4O	-2.749	0.000	Down
PE	PE 34:2,	-2.718	0.000	Down
PE	PE 36:3_PE 18:1_18:2,	-2.655	0.000	Down
PE	PE 34:0	-2.585	0.000	Down
PE	PE 36:3	-2.585	0.000	Down
PE	PE 36:3_PE 18:1_18:2	-2.583	0.000	Down
PE	PE P-34:2_PE P-16:0_18:2	-2.338	0.000	Down
PE	PE P-36:3_PE P-18:1_18:2	-2.336	0.000	Down
PE	PE O-36:3_PE O-18:2_18:1	-2.273	0.000	Down
PE	PE 36:3_PE 18:0_18:3	-2.143	0.002	Down
PE	PE 34:0_PE 16:0_18:0	-2.141	0.000	Down
PE	PE O-34:3_PE O-16:1_18:2	-2.123	0.001	Down
PE	PE 36:2	-2.109	0.000	Down
PE	PE 36:2;O_PE 18:0_18:2;O	-1.998	0.000	Down
PE	PE O-24:5_16:0	-1.894	0.000	Down
PE	PE 36:2_PE 18:1_18:1,	-1.875	0.000	Down
PE	PE P-38:5_PE P-18:1_20:4	-1.832	0.002	Down
PE	PE O-19:0_28:6	-1.827	0.009	Down
PE	PE O-22:4_22:6;3O	-1.824	0.000	Down
PE	PE O-36:2_PE O-18:1_18:1	-1.758	0.000	Down
PE	PE 38:5	-1.756	0.000	Down
PE	PE 40:5	-1.698	0.001	Down
PE	PE 36:2_PE 18:0_18:2	-1.676	0.002	Down
PE	PE 36:1_PE 18:0_18:1	-1.639	0.000	Down
PE	PE 36:2_PE 18:1_18:1	-1.638	0.002	Down
PE	PE 36:1_PE 18:0_18:1,	-1.506	0.001	Down
PE	PE O-38:6_PE O-18:2_20:4	-1.477	0.028	Down
PE	PE O-26:5_16:0	-1.437	0.000	Down
PE	PE 20:4_22:5	-1.390	0.004	Down
PE	PE 38:3	-1.345	0.002	Down
PE	PE O-37:4	-1.289	0.001	Down
PE-Cer	PE-Cer 13:1;2O_30:1	-3.359	0.000	Down
PE-Cer	PE-Cer 12:1;2O_16:1	-2.786	0.000	Down
PE-Cer	PE-Cer 12:1;2O_32:0	-1.781	0.000	Down
PE-Cer	PE-Cer 17:1;2O_36:8;O	-1.701	0.000	Down
PEtOH	PEtOH 26:2_PEtOH 13:1_13:1	-2.658	0.000	Down

PG	PG 28:0_8:0	-2.988	0.000	Down
PG	PG O-15:0_28:0	-2.901	0.000	Down
PG	PG 18:2_18:1;1O	-1.349	0.004	Down
PI-Cer	PI-Cer 39:4;3O	-3.259	0.000	Down
PI-Cer	PI-Cer 13:2;2O_22:6;O	-3.216	0.000	Down
PI-Cer	PI O-11:0_28:6	-3.170	0.000	Down
PI-Cer	PI 36:0	-3.038	0.000	Down
PI-Cer	PI 17:0_22:3;2O	-2.999	0.000	Down
PI-Cer	PI-Cer 13:1;2O_32:7;O	-2.967	0.000	Down
PI-Cer	PI-Cer 39:1;3O	-2.964	0.000	Down
PI-Cer	PI 34:1,	-2.938	0.000	Down
PI-Cer	PI 34:1	-2.880	0.000	Down
PI-Cer	PI 34:0	-2.848	0.000	Down
PI-Cer	PI 24:0_18:2;3O	-2.367	0.000	Down
PI-Cer	PI 18:0_28:6	-2.159	0.000	Down
PI-Cer	PI 18:0_28:5	-2.114	0.000	Down
PI-Cer	PI 36:1	-2.060	0.000	Down
PI-Cer	PI-Cer 36:2;3O	-2.020	0.001	Down
PI-Cer	PI 36:2,	-2.001	0.000	Down
PI-Cer	PI 24:0_22:6;4O	-1.979	0.000	Down
PI-Cer	PI 26:0_17:0;2O	-1.840	0.000	Down
PI-Cer	PI 16:0_28:5	-1.804	0.000	Down
PI-Cer	PI O-13:1_26:7	-1.772	0.000	Down
PI-Cer	PI 24:0_18:1;4O	-1.708	0.000	Down
PI-Cer	PI 20:5_24:0;1O	-1.707	0.001	Down
PI-Cer	PI 36:1_PI 18:0_18:1	-1.623	0.000	Down
PI-Cer	PI 16:0_22:6;4O	-1.588	0.000	Down
PI-Cer	PI 36:2	-1.482	0.001	Down
PMeOH	PMeOH 28:7_28:7	-2.785	0.000	Down
PS	PS 34:0	-3.176	0.000	Down
PS	PS 22:5_22:6;4O	-2.977	0.000	Down
PS	PS 8:0_28:1	-2.615	0.000	Down
PS	PS 44:2	-2.311	0.000	Down
PS	PS 36:2	-1.867	0.000	Down
PS	PS 36:2_PS 18:0_18:2	-1.792	0.000	Down
PS	PS 36:2,	-1.570	0.001	Down
PS	PS 36:1_PS 18:0_18:1	-1.515	0.000	Down
PS	PS 36:2_PS 18:0_18:2,	-1.429	0.002	Down
PS	PS 36:3	-1.416	0.007	Down
PS	PS 22:6_22:6	-1.345	0.015	Down
PS	PS 38:2	-1.242	0.002	Down
SE	SE 28:2_16:0	-4.088	0.000	Down
SE	SE 29:2_28:0	-3.796	0.000	Down
SE	SE 29:2_38:5	-3.795	0.000	Down
SE	SE 28:2_35:0	-3.765	0.000	Down
SE	SE 28:2_28:0	-3.744	0.000	Down
SE	SE 29:2_20:0	-3.735	0.000	Down
SE	SE 29:2_26:0	-3.733	0.000	Down

SE	SE 28:2_34:5	-3.693	0.000	Down
SE	SE 28:2_42:5	-3.687	0.000	Down
SE	SE 28:2_30:0	-3.679	0.000	Down
SE	SE 29:2_34:5	-3.664	0.000	Down
SE	SE 29:2_30:0	-3.661	0.000	Down
SE	SE 28:2_13:1	-3.649	0.000	Down
SE	SE 28:2_20:0	-3.638	0.000	Down
SE	SE 29:2_23:0	-3.632	0.000	Down
SE	SE 28:2_32:0	-3.626	0.000	Down
SE	SE 29:2_22:0	-3.613	0.000	Down
SE	SE 29:2_24:0	-3.590	0.000	Down
SE	SE 27:2_16:0	-3.574	0.000	Down
SE	SE 28:2_10:0	-3.538	0.000	Down
SE	SE 28:2_36:5	-3.481	0.000	Down
SE	SE 28:4_32:6	-2.374	0.000	Down
SE	SE 28:2_19:4	-2.228	0.000	Down
SE	SE 28:2_19:5	-1.667	0.000	Down
SHexCe	SHexCer 39:0;2O	-3.175	0.000	Down
SHexCe	SHexCer 32:0;3O	-2.736	0.000	Down
SHexCe	SHexCer 38:3;3O	-1.841	0.000	Down
SHexCe	SHexCer 43:3;3O	-1.835	0.000	Down
SHexCe	SHexCer 35:0;3O	-1.689	0.002	Down
SHexCe	SHexCer 12:1;2O_26:1	-1.417	0.000	Down
SL	SL 16:3;O_36:1;O	-3.366	0.000	Down
SL	SL 12:1;O_34:6	-3.362	0.000	Down
SL	SL 12:2;O_34:0;O	-3.263	0.000	Down
SL	SL 13:2;O_36:5;O	-2.826	0.000	Down
SL	SL 13:2;O_32:2;O	-2.724	0.000	Down
SL	SHexCer 45:4;3O	-1.954	0.000	Down
SL	SL 16:3;O_30:8	-1.529	0.017	Down
SL	SL 22:1;O_36:9	-1.212	0.001	Down
SL	SL 13:1;O_34:5	2.147	0.044	UP
SM	SM 42:0;2O	-4.491	0.000	Down
SM	SM 41:0;2O	-4.265	0.000	Down
SM	SM 42:1;2O	-3.855	0.000	Down
SM	SM 40:0;2O_SM 28:0;2O_12:0	-3.419	0.000	Down
SM	SM 21:0;3O	-3.134	0.000	Down
SM	SM 41:1;2O_SM 18:1;2O_23:0	-3.131	0.000	Down
SM	SM 42:1;3O	-2.981	0.000	Down
SM	SM 42:2;2O	-2.847	0.000	Down
SM	SM 42:2;2O_SM 18:1;2O_24:1	-2.847	0.000	Down
SM	SM 32:0;2O_SM 23:0;2O_9:0	-2.773	0.000	Down
SM	SM 32:0;2O	-2.697	0.000	Down
SM	SM 39:1;3O	-2.677	0.000	Down
SM	SM 42:1;2O_SM 18:1;2O_24:0	-2.615	0.000	Down
SM	SM 12:0;2O_27:0	-2.590	0.000	Down
SM	SM 15:3;2O_30:3	-2.483	0.001	Down
SM	SM 39:0;2O	-2.456	0.000	Down

SM	SM 42:1;2O	-2.404	0.000	Down
SM	SM 42:2;3O	-2.401	0.000	Down
SM	SM 40:1;2O_SM 18:1;2O_22:0	-2.381	0.000	Down
SM	SM 40:1;2O,	-2.355	0.000	Down
SM	SM 44:2;2O_SM 12:1;2O_32:1	-2.334	0.000	Down
SM	SM 41:4;3O	-2.272	0.000	Down
SM	SM 28:5;2O(FA 22:6)	-2.200	0.000	Down
SM	SM 42:3;2O_SM 18:1;2O_24:2	-2.023	0.000	Down
SM	SM 44:2;2O	-2.013	0.000	Down
SM	SM 44:3;2O	-2.004	0.000	Down
SM	SM 13:1;2O_28:1	-1.969	0.000	Down
SM	SM 42:4;2O	-1.938	0.000	Down
SM	SM 35:0;2O	-1.907	0.000	Down
SM	SM 44:1;2O	-1.852	0.000	Down
SM	SM 40:2;2O,	-1.834	0.000	Down
SM	SM 32:1;2O	-1.807	0.000	Down
SM	SM 42:3;3O,	-1.750	0.004	Down
SM	SM 42:3;3O	-1.738	0.001	Down
SM	SM 41:0;3O	-1.723	0.000	Down
SM	SM 12:1;2O_29:0	-1.694	0.000	Down
SM	SM 13:1;2O_28:2	-1.647	0.001	Down
SM	SM 38:0;2O	-1.633	0.001	Down
SM	SM 12:1;2O_28:3	-1.618	0.000	Down
SM	SM 12:0;2O_25:0	-1.554	0.001	Down
SM	SM 25:3;2O(FA 20:5)	-1.538	0.001	Down
SM	SM 32:1;2O_SM 17:0;2O_15:1	-1.532	0.000	Down
SM	SM 40:1;2O	-1.527	0.001	Down
SM	SM 39:0;3O	-1.491	0.001	Down
SM	SM 34:0;2O	-1.469	0.001	Down
SM	SM 40:2;2O	-1.404	0.000	Down
SM	SM 30:3;2O(FA 22:6)	-1.306	0.001	Down
SM	SM 12:1;2O_26:3	-1.271	0.001	Down
SM	SM 34:1;2O	-1.228	0.000	Down
SM	SM 36:0;2O_SM 28:0;2O_8:0	-1.023	0.013	Down
SMGDG	SMGDG O-21:4_28:7	-2.260	0.000	Down
SMGDG	SMGDG O-17:0_28:5	-1.511	0.000	Down
ST	ST 29:2;O;Hex;FA 20:1	-3.726	0.000	Down
ST	ST 24:1;O4_19:2;1O	-3.072	0.000	Down
ST	ST 24:2;O4_2:0	-2.773	0.000	Down
ST	SMGDG O-9:0_26:1	-2.764	0.000	Down
ST	ST 24:1;O4;G_16:2;1O	-2.554	0.000	Down
ST	ST 29:1;O;Hex;FA 15:2	-1.941	0.001	Down
ST	ST 24:1;O3;G_28:6	-1.776	0.026	Down
ST	ST 29:1;O;Hex;FA 13:0	-1.542	0.000	Down
ST	ST 24:1;O4;T_21:1	-1.485	0.000	Down
ST	ST 24:1;O3_23:0	-1.097	0.002	Down
TG	TG 49:2;1O_TG 16:0_16:0_17:2;1O	-3.144	0.000	Down
TG	TG 49:2_TG 16:0_16:1_17:1	-3.029	0.000	Down

TG	TG 49:2_TG 15:0_16:1_18:1	-2.743	0.000	Down
TG	TG 44:0_TG 14:0_14:0_16:0,	-2.533	0.000	Down
TG	TG O-41:0_TG O-11:0_14:0_16:0	-2.476	0.000	Down
TG	TG 46:1_TG 14:0_14:0_18:1	-2.452	0.000	Down
TG	TG 38:1_TG 10:0_10:0_18:1	-2.443	0.000	Down
TG	TG 44:1_TG 10:0_16:0_18:1	-2.439	0.000	Down
TG	TG 43:0_TG 13:0_14:0_16:0	-2.434	0.000	Down
TG	TG 46:1_TG 14:0_16:0_16:1	-2.425	0.000	Down
TG	TG 36:0_TG 10:0_12:0_14:0	-2.405	0.000	Down
TG	TG 36:0_TG 10:0_12:0_14:0,	-2.401	0.000	Down
TG	TG 47:0_TG 15:0_16:0_16:0,	-2.394	0.000	Down
TG	TG 46:0_TG 14:0_16:0_16:0	-2.392	0.000	Down
TG	TG O-52:1_TG O-19:1_16:0_17:0	-2.380	0.000	Down
TG	TG 44:0_TG 14:0_14:0_16:0	-2.374	0.000	Down
TG	TG 56:0;1O_TG 22:0_22:0_12:0;1O	-2.358	0.000	Down
TG	TG 45:1_TG 14:0_15:0_16:1	-2.357	0.000	Down
TG	TG 42:0_TG 12:0_14:0_16:0	-2.335	0.000	Down
TG	TG 38:1;1O_TG 8:0_16:0_14:1;1O	-2.334	0.000	Down
TG	TG 47:1_TG 15:0_16:0_16:1	-2.330	0.000	Down
TG	TG 40:0_TG 12:0_12:0_16:0	-2.326	0.000	Down
TG	TG 45:0_TG 15:0_15:0_15:0	-2.317	0.000	Down
TG	TG 40:0_TG 10:0_14:0_16:0	-2.262	0.000	Down
TG	TG 46:0_TG 14:0_16:0_16:0,	-2.215	0.000	Down
TG	TG 42:0_TG 12:0_14:0_16:0,	-2.208	0.000	Down
TG	TG 52:1;3O_TG 17:0_17:0_18:1;3O	-2.189	0.000	Down
TG	TG 43:0_TG 12:0_15:0_16:0	-2.177	0.000	Down
TG	TG 49:0_TG 15:0_17:0_17:0	-2.165	0.000	Down
TG	TG 38:0_TG 8:0_14:0_16:0	-2.147	0.000	Down
TG	TG 48:0_TG 16:0_16:0_16:0,	-2.105	0.000	Down
TG	TG 38:0_TG 12:0_12:0_14:0	-2.071	0.000	Down
TG	TG 38:1_TG 10:0_14:0_14:1	-2.043	0.000	Down
TG	TG 40:1_TG 8:0_16:0_16:1	-1.983	0.000	Down
TG	TG 48:2_TG 16:0_16:1_16:1	-1.961	0.000	Down
TG	TG O-54:1_TG O-18:0_18:0_18:1	-1.953	0.000	Down
TG	TG 49:0_TG 16:0_16:0_17:0	-1.911	0.000	Down
TG	TG 42:2_TG 10:0_14:1_18:1	-1.910	0.000	Down
TG	TG 38:1_TG 10:0_10:0_18:1,	-1.881	0.000	Down
TG	TG 56:0_TG 15:0_16:0_25:0	-1.862	0.000	Down
TG	TG O-57:1_TG O-19:0_16:1_22:0	-1.860	0.000	Down
TG	TG 49:0_TG 16:0_16:0_17:0,	-1.780	0.000	Down
TG	TG 48:4_TG 14:0_16:1_18:3	-1.780	0.000	Down
TG	TG 48:1_TG 14:0_16:0_18:1	-1.776	0.000	Down
TG	TG 36:0_TG 12:0_12:0_12:0	-1.764	0.000	Down
TG	TG 47:0_TG 15:0_16:0_16:0	-1.761	0.000	Down
TG	TG 42:1_TG 10:0_14:0_18:1	-1.745	0.000	Down
TG	TG 40:2_TG 12:0_14:1_14:1	-1.726	0.000	Down
TG	TG 54:0_TG 18:0_18:0_18:0	-1.704	0.000	Down
TG	TG 58:0_TG 16:0_16:0_26:0	-1.679	0.000	Down

TG	TG O-55:1_TG O-19:1_18:0_18:0	-1.634	0.000	Down
TG	TG 48:3_TG 14:0_16:1_18:2,	-1.575	0.000	Down
TG	TG 55:0_TG 15:0_16:0_24:0	-1.562	0.000	Down
TG	TG 55:2_TG 18:0_18:1_19:1	-1.555	0.000	Down
TG	TG 48:0_TG 16:0_16:0_16:0	-1.554	0.000	Down
TG	TG 49:1_TG 16:0_16:0_17:1	-1.537	0.000	Down
TG	TG 51:0_TG 17:0_17:0_17:0	-1.509	0.000	Down
TG	TG 8:0_9:0_22:1	-1.503	0.000	Down
TG	TG 47:1;1O_TG 16:0_16:0_15:1;1O	-1.481	0.000	Down
TG	TG 51:0_TG 16:0_17:0_18:0	-1.410	0.000	Down
TG	TG 38:0_TG 8:0_12:0_18:0	-1.382	0.001	Down
TG	TG 54:0_TG 18:0_18:0_18:0,	-1.359	0.000	Down
TG	TG 53:0_TG 14:0_16:0_23:0	-1.336	0.000	Down
TG	TG 48:2_TG 14:0_16:1_18:1	-1.331	0.001	Down
TG	TG 50:0_TG 16:0_16:0_18:0,	-1.168	0.003	Down
TG	TG 50:2_TG 16:0_16:1_18:1,	-1.111	0.001	Down
TG	TG 56:1_TG 16:0_22:0_18:1	-1.100	0.003	Down
TG	TG 48:3_TG 14:0_16:1_18:2	-1.033	0.004	Down
TG	TG 34:0_TG 8:0_12:0_14:0	-1.028	0.001	Down
TG	TG O-49:1_TG O-17:1_14:0_18:0	-1.012	0.002	Down
TG	TG 54:3;1O_TG 18:1_18:1_18:1;1O	1.629	0.011	UP
TG	TG 54:4_TG 18:1_18:1_18:2	2.808	0.013	UP
