# Supplementary material for: Untargeted lipidomics reveals unique lipid signatures of extracellular vesicles from porcine colostrum and milk
Source: PLoS One. 2025 Feb 13;20(2):e0313683. doi: 10.1371/journal.pone.0313683 (PMC11825007; doi:10.1371/journal.pone.0313683)
Supplement: S4 Table — https://doi.org/10.6084/m9.figshare.28016414.v1. (PDF) [file pone.0313683.s008.pdf]

S4 Table. List of all significantly up- and down-regulated lipids elements in comparison between porcine colostrum exosomes (day 0) and milk exosomes at day 14. Significance was set using t-test FDR adjusted p-value threshold at 0.05 and fold change threshold at 2 (| $\log_2$ FC > 1 |).

Day 0 vs 14				
**Lipid class**	**Lipid name**	**log2(FC)**	**FDR Ajusted P value**	**UP/Down**
SL	SL 12:1;O_34:6	-12.586	0.002	Down
Cer	Cer 35:5;5O_Cer 19:2;3O_16:3;(2OH)	-11.829	0.002	Down
SM	SM 32:0;2O	-11.528	0.002	Down
SM	SM 34:1;2O	-10.275	0.048	Down
MAE	NAE 16:2	-10.194	0.005	Down
PC	PC 32:0,	-10.109	0.002	Down
DG	DG 35:6	-10.078	0.000	Down
Cer	Cer 94:5;4O	-9.599	0.003	Down
DG	DG 42:0	-9.590	0.000	Down
MG	MGDG O-16:4_22:6	-9.505	0.001	Down
AHexCer	AHexCer 60:8;3O	-9.343	0.003	Down
DG	DG 49:6	-9.321	0.000	Down
DG	DG 43:0	-9.297	0.000	Down
DG	DG 42:5	-9.281	0.000	Down
DG	DG O-55:1_DG O-27:0_28:1	-9.273	0.000	Down
DG	DG 45:0	-9.273	0.000	Down
SE	SE 28:2_30:0	-9.271	0.000	Down
SE	SE 29:2_28:0	-9.239	0.000	Down
DG	DG 86:5	-9.237	0.000	Down
SE	SE 28:2_28:0	-9.222	0.000	Down
Cer	Cer 12:0;2O_23:0;O	-9.217	0.000	Down
SM	SM 42:1;2O	-9.183	0.003	Down
DG	DG 35:0	-9.153	0.000	Down
SE	SE 29:2_26:0	-9.147	0.000	Down
SE	SE 28:2_32:0	-9.123	0.000	Down
AHexCer	AHexCer 72:9;3O	-9.079	0.004	Down
SE	SE 29:2_30:0	-9.077	0.000	Down
DG	DG 36:0	-9.067	0.000	Down
DG	DG 50:0	-9.041	0.000	Down
DG	DG O-48:1_DG O-20:0_28:1	-9.037	0.000	Down
SM	SM 12:1;2O_26:3	-9.012	0.020	Down
DG	DG 39:0	-9.006	0.000	Down
DG	DG 51:0	-8.987	0.000	Down
DG	DG 47:0	-8.965	0.000	Down
SM	SM 44:2;2O_SM 12:1;2O_32:1	-8.956	0.003	Down
DG	DG 46:0	-8.946	0.000	Down
SE	SE 29:2_23:0	-8.945	0.000	Down
DG	DG 40:0	-8.928	0.000	Down
SE	SE 28:2_34:5	-8.923	0.000	Down
MAE	NAE 18:5	-8.893	0.001	Down

DG	DG 19:0	-8.889	0.000	Down
SE	SE 29:2_24:0	-8.862	0.000	Down
SE	SE 29:2_38:5	-8.852	0.000	Down
SE	SE 28:2_42:5	-8.851	0.000	Down
DG	DG 41:0	-8.846	0.000	Down
SE	SE 28:2_35:0	-8.842	0.000	Down
DG	DG 39:1	-8.816	0.000	Down
DG	DG 44:6_DG 16:0_28:6	-8.793	0.000	Down
DG	DG O-44:1_DG O-18:0_26:1	-8.793	0.000	Down
SE	SE 28:2_16:0	-8.786	0.000	Down
DG	DG 43:8	-8.769	0.000	Down
DG	DG 41:6	-8.768	0.000	Down
DG	DG 52:6	-8.756	0.000	Down
DG	DG 49:0	-8.747	0.000	Down
ST	ST 29:2;O;Hex;FA 20:1	-8.734	0.000	Down
MG	MG 13:0	-8.733	0.001	Down
DG	DG 46:11	-8.673	0.000	Down
DG	DG 51:7	-8.672	0.000	Down
SE	SE 29:2_22:0	-8.669	0.000	Down
DG	DG 50:6	-8.661	0.000	Down
SE	SE 28:2_10:0	-8.611	0.000	Down
DG	DG O-36:1_DG O-17:0_19:1	-8.610	0.000	Down
DG	DG 49:2	-8.590	0.000	Down
MG	MG 18:3	-8.577	0.000	Down
DG	DG O-38:1_DG O-14:0_24:1	-8.565	0.000	Down
SM	SM 12:1;2O_28:3	-8.565	0.005	Down
DG	DGCC 36:2_DGCC 18:1_18:1	-8.543	0.000	Down
DG	DG 44:0	-8.541	0.000	Down
DG	DG 30:5	-8.496	0.000	Down
DG	DG O-30:1_DG O-16:0_14:1	-8.481	0.000	Down
SM	SM 42:0;2O	-8.472	0.000	Down
DG	DG O-33:1_DG O-17:0_16:1	-8.463	0.000	Down
SM	SM 36:0;2O_SM 28:0;2O_8:0	-8.445	0.021	Down
DG	DG 39:6	-8.396	0.000	Down
DG	DG 27:5	-8.371	0.000	Down
CAR	CAR 16:0	-8.368	0.005	Down
MG	MG 22:5	-8.353	0.000	Down
SM	SM 41:1;2O_SM 18:1;2O_23:0	-8.350	0.000	Down
Cer	Cer 94:3;4O	-8.335	0.000	Down
DG	DG 47:6	-8.313	0.000	Down
PS	PS 36:2	-8.286	0.002	Down
SE	SE 27:2_16:0	-8.281	0.000	Down
DG	DG 45:7	-8.239	0.000	Down
Cer	Cer 36:1;3O_Cer 19:0;2O_17:1;O	-8.226	0.000	Down
DG	DG 27:4	-8.207	0.000	Down
SE	SE 29:2_20:0	-8.195	0.000	Down
DG	DG 43:7	-8.183	0.000	Down

AHexCer	AHexCer 54:6;3O	-8.167	0.007	Down
DG	DG 46:6	-8.159	0.000	Down
DG	DG 28:2	-8.154	0.000	Down
DG	DG 41:6_DG 15:0_26:6	-8.148	0.000	Down
SM	SM 41:0;2O	-8.137	0.000	Down
DG	DG 45:8	-8.129	0.000	Down
DG	DG 38:6	-8.120	0.000	Down
DG	DG 48:6	-8.105	0.000	Down
DG	DG 40:6	-8.084	0.000	Down
MAE	NAE 14:1	-8.028	0.000	Down
MAE	NAE 15:4	-8.015	0.000	Down
DG	DG 49:8	-8.013	0.000	Down
Cer	Cer 92:5;4O	-8.001	0.002	Down
DG	DG 20:0	-7.970	0.000	Down
PS	PS 38:2	-7.970	0.017	Down
DG	DG O-41:1_DG O-13:0_28:1	-7.947	0.000	Down
DG	DG 49:7	-7.916	0.000	Down
MG	MG 16:3	-7.901	0.000	Down
DG	DG 52:7	-7.898	0.000	Down
DG	DG O-39:1_DG O-13:0_26:1	-7.883	0.000	Down
AHexCer	AHexCer 40:4;3O	-7.881	0.000	Down
SE	SE 28:2_20:0	-7.873	0.000	Down
PS	PS 36:3	-7.848	0.003	Down
DG	DG O-40:1_DG O-16:0_24:1	-7.846	0.000	Down
SE	SE 28:4_32:6	-7.812	0.001	Down
Cer	Cer 65:5;4O	-7.808	0.000	Down
SE	SE 29:2_34:5	-7.779	0.000	Down
DG	DG 45:6	-7.749	0.000	Down
DG	DG 42:6_DG 16:0_26:6	-7.740	0.000	Down
DG	DG 41:7	-7.712	0.000	Down
PC	PC 10:0_26:1	-7.712	0.010	Down
Cer	Cer 61:13;4O	-7.694	0.002	Down
DG	DG 25:1	-7.690	0.000	Down
DG	DG 51:6	-7.681	0.000	Down
MAE	NAE 18:4	-7.636	0.000	Down
DG	DG 39:8	-7.630	0.000	Down
Cer	Cer 38:3;5O_Cer 21:2;3O_17:1;(2OH)	-7.615	0.000	Down
DG	DG 51:4	-7.545	0.000	Down
PC	PC 34:3	-7.518	0.001	Down
Cer	Cer 13:2;2O_40:3	-7.509	0.000	Down
MAE	NAE 14:0	-7.493	0.000	Down
DG	DG 51:13	-7.492	0.004	Down
PE	PE 32:1	-7.469	0.000	Down
DG	DG 51:8	-7.466	0.000	Down
DG	DG 43:6	-7.465	0.000	Down
DG	DG 36:0_DG 18:0_18:0	-7.447	0.000	Down
SE	SE 28:2_36:5	-7.430	0.000	Down

DG	DG 50:1	-7.413	0.000	Down
DG	DG 36:6	-7.412	0.000	Down
Cer	Cer 38:5;4O	-7.405	0.000	Down
SE	SE 28:2_13:1	-7.358	0.000	Down
CE	CE 18:1	-7.338	0.000	Down
DG	DG 18:0	-7.331	0.001	Down
DG	DG 36:1_DG 18:0_18:1	-7.307	0.000	Down
PC	PC O-36:7	-7.243	0.000	Down
DG	DG 31:0	-7.232	0.000	Down
MAE	NAE 16:4	-7.224	0.000	Down
Cer	CerP 29:2;2O_CerP 16:1;2O_13:1	-7.220	0.000	Down
SM	SM 42:1;2O	-7.205	0.000	Down
Cer	Cer 58:5;4O	-7.130	0.001	Down
MG	MG 15:0	-7.127	0.000	Down
DG	DG 34:0	-7.122	0.000	Down
SM	SM 40:2;2O,	-7.106	0.003	Down
DG	DG 22:1	-7.096	0.000	Down
DG	DG 37:4	-7.083	0.000	Down
DG	DG 39:7	-7.077	0.000	Down
Cer	Cer 36:2;3O_Cer 19:0;2O_17:2;O	-7.061	0.000	Down
DG	DG 25:2	-7.043	0.000	Down
MAE	NAE 26:5	-7.029	0.000	Down
DG	DG 42:10	-7.026	0.000	Down
MG	MG 21:1	-7.015	0.000	Down
Cer	Cer 40:0;4O_Cer 30:0;3O_10:0;(2OH)	-7.008	0.000	Down
DG	DG 25:0	-7.007	0.000	Down
DG	DG 37:7	-7.005	0.000	Down
DG	DG 34:0_DG 16:0_18:0	-6.994	0.000	Down
DG	DG O-35:0_DG O-19:0_16:0	-6.986	0.000	Down
DG	DG 34:2_DG 16:0_18:2	-6.980	0.000	Down
CAR	CAR 18:1	-6.957	0.012	Down
DG	DG 16:0	-6.946	0.000	Down
PI	PI 34:1	-6.944	0.000	Down
MAE	NAE 22:3	-6.940	0.000	Down
Cer	Cer 34:1;3O_Cer 19:0;2O_15:1;O	-6.927	0.000	Down
DG	DG 34:3	-6.926	0.000	Down
DG	DG 36:5	-6.924	0.000	Down
PC	PC 38:5,	-6.909	0.028	Down
SM	SM 42:1;3O	-6.894	0.001	Down
DG	DG 27:0	-6.892	0.000	Down
Cer	Cer 12:2;2O_24:1	-6.889	0.000	Down
DG	DG 36:2	-6.882	0.000	Down
DG	DG O-45:6_DG O-17:0_28:6	-6.852	0.000	Down
MAE	NAE 19:5	-6.846	0.000	Down
DG	DG 43:2	-6.824	0.000	Down
Cer	CerP 34:2;2O_CerP 19:1;2O_15:1	-6.823	0.000	Down
MAE	NAE 18:3	-6.758	0.000	Down

DG	DG 40:2	-6.751	0.000	Down
ST	ST 24:1;O4_19:2;1O	-6.729	0.000	Down
DG	DG 38:5	-6.725	0.000	Down
DG	DG 40:5	-6.713	0.000	Down
DG	DG 32:0	-6.692	0.000	Down
HexCer	HexCer 34:1;3O_HexCer 18:1;2O_16:0;O	-6.685	0.004	Down
Cer	Cer 12:2;2O_39:10;2O	-6.673	0.000	Down
ST	ST 24:1;O4;T_26:2	-6.668	0.011	Down
PC	PC O-39:0	-6.668	0.000	Down
DG	DG 34:2	-6.658	0.000	Down
DG	DG 55:9	-6.652	0.007	Down
DG	DGTS 16:0_17:3	-6.642	0.000	Down
DG	DG O-42:6_DG O-18:0_24:6	-6.623	0.000	Down
DG	DG 38:2	-6.603	0.000	Down
DG	DG 53:10	-6.589	0.005	Down
DG	DG 41:8	-6.575	0.002	Down
DG	DG 52:11	-6.541	0.000	Down
PC	PC O-35:7	-6.534	0.000	Down
Cer	Cer 60:11;4O	-6.519	0.000	Down
CAR	CAR 19:3	-6.517	0.000	Down
Cer	Cer 81:9;4O	-6.516	0.022	Down
Cer	Cer 36:4;4O	-6.506	0.000	Down
MAE	NAE 26:6	-6.497	0.000	Down
PE	PE 34:2	-6.485	0.000	Down
SM	SM 32:1;2O	-6.483	0.001	Down
SM	SM 40:0;2O_SM 28:0;2O_12:0	-6.479	0.000	Down
DG	DG 36:1	-6.476	0.000	Down
MAE	NAE 21:4	-6.463	0.000	Down
DG	DG 46:8	-6.456	0.000	Down
SM	SM 39:0;2O	-6.445	0.000	Down
PC	PC 30:1	-6.441	0.000	Down
DG	DG 24:1	-6.433	0.000	Down
DG	DG 32:3	-6.430	0.000	Down
DG	DG 33:1	-6.418	0.000	Down
DG	DG 46:3	-6.416	0.000	Down
DG	DGCC 15:2_18:5	-6.414	0.000	Down
DG	DG 29:0	-6.382	0.000	Down
Cer	Cer 38:0;2O_Cer 18:0;2O_20:0	-6.380	0.000	Down
MAE	NAE 20:3	-6.379	0.000	Down
DG	DG 26:0	-6.355	0.000	Down
DG	DG 52:13	-6.349	0.012	Down
DG	DG 30:0	-6.331	0.000	Down
Cer	CerP 16:2;2O_28:3	-6.312	0.042	Down
PS	PS 34:0	-6.311	0.000	Down
Cer	Cer 58:11;4O	-6.310	0.000	Down
DG	DG 32:0_DG 16:0_16:0	-6.290	0.000	Down
DG	DG 34:1	-6.281	0.001	Down

Cer	Cer 35:6;2O_Cer 17:3;2O_18:3	-6.276	0.000	Down
Cer	Cer 31:4;2O_Cer 12:2;2O_19:2	-6.270	0.001	Down
MAE	NAE 24:5	-6.258	0.000	Down
DG	DG 23:4	-6.207	0.000	Down
DG	DG 28:1	-6.191	0.001	Down
MG	MG 19:5	-6.145	0.000	Down
ST	ST 24:2;O4_2:0	-6.137	0.000	Down
SE	SE 28:2_19:4	-6.116	0.001	Down
DG	DG 28:0	-6.112	0.000	Down
SL	SL 22:1;O_36:9	-6.111	DDG	Down
MAE	NAE 20:5	-6.089	0.000	Down
DG	DG 29:5	-6.061	0.000	Down
MAE	NAE 15:1	-6.054	0.000	Down
CoQ10	CoQ10	-6.030	0.001	Down
CAR	CAR 13:0	-6.027	0.000	Down
PC	PC O-32:1	-5.997	0.000	Down
NAGly	NAGly 21:1_9:0	-5.978	0.000	Down
DG	DG 32:2	-5.958	0.000	Down
Cer	Cer 28:3;4O,	-5.943	0.000	Down
SM	SM 42:2;3O	-5.932	0.009	Down
SM	SM 44:3;2O	-5.929	0.000	Down
DG	DG 31:4	-5.909	0.000	Down
DG	DG 45:11	-5.894	0.000	Down
SHexCer	SHexCer 12:1;2O_26:1	-5.870	0.023	Down
Cer	Cer 50:10;4O	-5.861	0.000	Down
DG	DG 40:8	-5.826	0.000	Down
Cer	Cer 12:1;2O_30:0	-5.824	0.001	Down
Cer	Cer 42:1;2O_Cer 18:1;2O_24:0,	-5.804	0.001	Down
PC	PC 33:1	-5.803	0.000	Down
DG	DG 40:9	-5.776	0.000	Down
DG	DG 53:9	-5.766	0.001	Down
CAR	CAR 5:0	-5.740	0.000	Down
DG	DG 24:2	-5.730	0.000	Down
SE	SE 28:2_19:5	-5.728	0.001	Down
DG	DG 44:7	-5.713	0.000	Down
MG	MG 17:0	-5.701	0.000	Down
Cer	Cer 34:3;4O_Cer 19:2;3O_15:1;(2OH)	-5.694	0.000	Down
PC	PC 32:2_PC 16:1_16:1	-5.692	0.000	Down
Cer	CerP 30:3;2O_CerP 12:0;2O_18:3	-5.678	0.000	Down
Cer	Cer 42:2;2O_Cer 18:1;2O_24:1	-5.668	0.003	Down
HexCer	HexCer 34:0;2O	-5.642	0.004	Down
DG	DGGA 12:0_22:1	-5.640	0.001	Down
DG	DG 57:9	-5.636	0.016	Down
DG	DGGA 10:0_22:1	-5.625	0.000	Down
DG	DG 36:4	-5.618	0.000	Down
PC	PC 28:0_PC 12:0_16:0	-5.617	0.000	Down
Cer	Cer 89:6;4O	-5.598	0.018	Down

Cer	Cer 42:0;4O	-5.593	0.000	Down
DG	DG 23:1	-5.575	0.000	Down
MAE	NAE 16:0	-5.572	0.000	Down
PE	PE 32:1_PE 16:0_16:1	-5.571	0.000	Down
PE	PE O-32:2_PE O-16:1_16:1	-5.544	0.000	Down
NAGly	NAGly 22:6_21:5	-5.542	0.000	Down
MAE	NAE 13:1	-5.531	0.000	Down
PS	PS 8:0_28:1	-5.502	0.000	Down
Cer	Cer 42:1;2O_Cer 18:1;2O_24:0	-5.487	0.001	Down
DG	DG 48:13	-5.481	0.000	Down
SM	SM 42:4;2O	-5.472	0.000	Down
Cer	Cer 38:1;2O_Cer 18:1;2O_20:0	-5.468	0.012	Down
MAE	NAE 15:0	-5.466	0.000	Down
MAE	NAE 20:4	-5.445	0.000	Down
PS	PS 36:1_PS 18:0_18:1	-5.434	0.004	Down
PE	PE 34:3	-5.424	0.000	Down
AHexCer	AHexCer 72:4;3O	-5.421	0.036	Down
MG	MG 9:0	-5.404	0.000	Down
PS	PS 36:2_PS 18:0_18:2	-5.399	0.001	Down
DG	DG 34:1_DG 16:0_18:1	-5.386	0.000	Down
DG	DG 35:2	-5.378	0.000	Down
DG	DG 34:5	-5.356	0.000	Down
Cer	Cer 28:3;4O	-5.351	0.000	Down
MAE	NAE 22:5	-5.340	0.000	Down
PE	PE 34:2,	-5.340	0.000	Down
AHexCer	AHexCer 39:4;3O_AHexCer (O-14:1)25:3;3O	-5.333	0.004	Down
MAE	NAE 16:1	-5.316	0.000	Down
DG	DG 23:0	-5.315	0.000	Down
MAE	NAE 20:1	-5.314	0.000	Down
DG	DG 23:2	-5.296	0.000	Down
DG	DG 32:7	-5.289	0.000	Down
LDGTS	LDGTS 15:0	-5.286	0.000	Down
SM	SM 30:3;2O(FA 22:6)	-5.282	0.003	Down
Cer	Cer 42:1;3O_Cer 18:1;2O_24:0;O	-5.274	0.003	Down
Cer	Cer 40:7;4O	-5.262	0.000	Down
PI	PI 36:1	-5.261	0.000	Down
DG	DG 38:8	-5.249	0.000	Down
DG	DG 33:7	-5.222	0.000	Down
SM	SM 44:2;2O	-5.215	0.001	Down
MG	MGDG O-14:1_20:4	-5.205	0.017	Down
FA	FA 16:2;3O	-5.202	0.000	Down
Cer	Cer 33:6;4O	-5.201	0.000	Down
Cer	Cer 24:3;3O_Cer 16:3;2O_8:0;O	-5.200	0.000	Down
PI	PI-Cer 39:1;3O	-5.191	0.000	Down
Cer	Cer 34:0;3O_Cer 18:0;2O_16:0;O	-5.186	0.000	Down
PS	PS 44:2	-5.179	0.001	Down
BMP	BMP 15:0_8:0	-5.152	0.000	Down

DG	DG 30:2	-5.143	0.000	Down
PC	PC 35:2	-5.142	0.001	Down
PC	PC 33:0	-5.135	0.001	Down
DG	DG 26:5	-5.128	0.000	Down
DG	DG 42:11	-5.126	0.000	Down
DG	DGDG O-8:0_17:0	-5.120	0.003	Down
PC	PC 38:6	-5.120	0.001	Down
PC	PC 32:1	-5.110	0.000	Down
MAE	NAE 15:3	-5.110	0.000	Down
DG	DG 38:7	-5.101	0.000	Down
MAE	NAE 17:4	-5.094	0.000	Down
DG	DG 26:2	-5.092	0.000	Down
DG	DG 38:4	-5.090	0.002	Down
DG	DG 36:2_DG 18:1_18:1	-5.088	0.000	Down
PI	PI-Cer 39:4;3O	-5.074	0.000	Down
DG	DG 29:3	-5.074	0.000	Down
NAGly	NAGly 30:0_NAGly 20:0_10:0	-5.068	0.000	Down
SM	SM 39:1;3O	-5.060	0.000	Down
DG	DG 32:6	-5.058	0.000	Down
DG	DG 27:2	-5.050	0.000	Down
DG	DG 30:3	-5.041	0.000	Down
AHexCer	AHexCer 59:9;3O	-5.029	0.000	Down
PC	PC 35:1	-5.026	0.002	Down
Cer	Cer 12:2;2O_30:0	-4.993	0.001	Down
SM	SM 44:1;2O	-4.989	0.003	Down
DG	DG 34:4	-4.960	0.000	Down
DG	DG 32:1	-4.951	0.000	Down
PC	PC O-30:0	-4.944	0.000	Down
FA	FA 44:5	-4.921	0.000	Down
PE	PE P-32:1_PE P-16:0_16:1	-4.917	0.000	Down
DG	DG 22:0	-4.907	0.000	Down
DG	DG 31:6	-4.898	0.000	Down
PC	PC O-37:8	-4.897	0.000	Down
FA	FA 40:5	-4.876	0.000	Down
FA	FA 20:4;3O	-4.859	0.000	Down
MG	MG 10:0	-4.858	0.000	Down
Cer	Cer 35:5;4O	-4.844	0.000	Down
SM	SM 42:2;2O_SM 18:1;2O_24:1	-4.839	0.000	Down
BMP	BMP 17:1_17:1	-4.834	0.000	Down
DG	DG 32:8	-4.821	0.000	Down
Cer	Cer 76:7;4O	-4.816	0.020	Down
ST	ST 24:1;O4;T_20:3;1O	-4.812	0.032	Down
DG	DG 36:3_DG 18:1_18:2	-4.810	0.000	Down
DG	DG 29:2	-4.803	0.000	Down
DG	DG 24:0	-4.803	0.000	Down
SL	SL 21:1;O_36:9;O	-4.796	0.031	Down
PC	PC 40:5	-4.795	0.003	Down

Cer	Cer 40:1;2O_Cer 18:1;2O_22:0,	-4.789	0.007	Down
PC	PC O-36:4	-4.780	0.000	Down
FA	FA 42:5	-4.773	0.000	Down
MAE	NAE 22:4	-4.772	0.000	Down
SM	SM 28:5;2O(FA 22:6)	-4.769	0.001	Down
DG	DG 29:4	-4.764	0.000	Down
Cer	CerP 14:1;2O_28:3	-4.742	0.006	Down
DG	DG 48:9	-4.737	0.025	Down
CAR	CAR 17:3	-4.733	0.000	Down
NAOrn	NAOrn 14:1;O	-4.721	0.000	Down
MAE	NAE 7:0	-4.717	0.000	Down
DG	DG 24:3	-4.708	0.000	Down
SM	SM 42:1;2O_SM 18:1;2O_24:0	-4.684	0.000	Down
DG	DG 30:8	-4.684	0.000	Down
CAR	CAR 7:0	-4.671	0.000	Down
DG	DG 30:7	-4.651	0.000	Down
DG	DG 39:10	-4.644	0.000	Down
DG	DG 31:8	-4.626	0.000	Down
MAE	NAE 18:1	-4.624	0.000	Down
NAOrn	NAOrn 13:0;O	-4.615	0.000	Down
Cer	Cer 33:5;4O	-4.613	0.000	Down
FA	FA 17:4;2O	-4.609	0.000	Down
Cer	Cer 12:1;2O_28:0	-4.608	0.004	Down
DG	DG 41:11	-4.606	0.000	Down
DG	DG 31:7	-4.599	0.000	Down
DG	DG 27:3	-4.593	0.000	Down
DG	DG 31:5	-4.589	0.000	Down
SM	SM 35:0;2O	-4.589	0.000	Down
MG	MG 12:0	-4.587	0.000	Down
SL	SL 18:1;O_36:4;O	-4.584	0.019	Down
MG	MG 17:4	-4.574	0.000	Down
SM	SM 38:2;2O	-4.567	0.008	Down
SM	SM 32:0;2O_SM 23:0;2O_9:0	-4.544	0.000	Down
PC	PC 34:2	-4.531	0.000	Down
NAGly	NAGly 13:1;O	-4.517	0.000	Down
DG	DG 28:5	-4.497	0.000	Down
SM	SM 41:4;3O	-4.491	0.000	Down
DG	DG 28:3	-4.485	0.000	Down
DG	DG 39:9	-4.469	0.000	Down
PC	PC 36:2	-4.455	0.004	Down
PC	PC O-30:7	-4.441	0.024	Down
SM	SM 34:0;3O	-4.435	0.009	Down
PC	PC O-39:3	-4.422	0.000	Down
DG	DG 28:4	-4.414	0.000	Down
DG	DG 30:4	-4.401	0.000	Down
FA	FA 16:1;3O	-4.401	0.000	Down
FA	FA 19:1;2O	-4.399	0.000	Down

SM	SM 38:0;2O	-4.394	0.001	Down
PC	PC 34:3_PC 16:1_18:2	-4.393	0.000	Down
PC	PC O-32:0	-4.383	0.000	Down
DG	DG 30:6	-4.362	0.000	Down
MAE	NAE 20:2	-4.361	0.000	Down
PE	PE O-26:6_14:0	-4.345	0.000	Down
HexCer	HexCer 16:1;3O_17:0;(2OH)	-4.339	0.000	Down
PC	PC 32:1_PC 16:0_16:1	-4.320	0.000	Down
HexCer	HexCer 18:1;2O_18:5	-4.308	0.000	Down
MAE	NAE 6:0	-4.294	0.000	Down
PE	PE 34:2_PE 16:1_18:1	-4.290	0.000	Down
PC	PC O-39:7	-4.288	0.016	Down
FA	FA 16:0;3O	-4.275	0.000	Down
SM	SM 21:0;3O	-4.259	0.000	Down
SM	SM 42:3;2O_SM 18:1;2O_24:2	-4.256	0.000	Down
FA	FA 42:9	-4.245	0.000	Down
PC	PC 36:1	-4.222	0.001	Down
Cer	Cer 40:1;2O_Cer 18:1;2O_22:0	-4.208	0.004	Down
DG	DG 52:10	-4.169	0.003	Down
DG	DG 49:1	-4.165	0.006	Down
DG	DGDG 8:0_20:2	-4.139	0.000	Down
PE	PE P-34:1_PE P-16:0_18:1	-4.124	0.000	Down
PC	PC 38:5	-4.119	0.011	Down
Cer	CerP 28:2;2O_CerP 13:1;2O_15:1	-4.119	0.028	Down
PC	PC O-38:7	-4.117	0.014	Down
DG	DG 52:14	-4.114	0.000	Down
FA	FA 16:1	-4.104	0.000	Down
DG	DGCC 15:0_22:6	-4.101	0.000	Down
DG	DGCC 16:0_19:5	-4.100	0.000	Down
PC	PC 36:2_PC 18:0_18:2	-4.074	0.009	Down
PE	PE P-36:2_PE P-18:1_18:1	-4.072	0.000	Down
PC	PC 30:0	-4.047	0.000	Down
LPC	LPC 28:7	-4.020	0.022	Down
MG	MG 15:4	-4.005	0.000	Down
FA	FA 15:4	-3.975	0.000	Down
MG	MG 15:2	-3.964	0.000	Down
SM	SM 38:1;2O	-3.943	0.023	Down
SM	SM 40:2;2O	-3.933	0.001	Down
PC	PC 36:0	-3.923	0.002	Down
SM	SM 40:1;2O_SM 18:1;2O_22:0	-3.921	0.000	Down
DG	DG 48:12	-3.894	0.000	Down
SHexCer	SHexCer 32:0;3O	-3.893	0.000	Down
PE	PE P-36:3_PE P-18:1_18:2	-3.891	0.000	Down
Cer	Cer 38:1;2O_Cer 18:1;2O_20:0,	-3.860	0.010	Down
FA	FA 38:5	-3.853	0.000	Down
NAOrn	NAOrn 22:2_20:0	-3.849	0.001	Down
PE	PE 34:2_PE 16:0_18:2	-3.826	0.000	Down

PE	PE 32:0_PE 16:0_16:0	-3.819	0.000	Down
MAE	NAE 16:3	-3.818	0.000	Down
PC	PC O-37:1	-3.793	0.000	Down
DG	DG 57:11	-3.788	0.047	Down
PE	PE O-37:4	-3.788	0.002	Down
PE	PE P-32:0_PE P-16:0_16:0	-3.788	0.000	Down
PC	PC O-34:1	-3.783	0.000	Down
PC	PC O-34:0	-3.781	0.000	Down
MG	MG 15:3	-3.774	0.000	Down
MAE	NAE 18:2	-3.771	0.000	Down
PI	PI 36:2	-3.749	0.001	Down
LPE	LPE O-17:1	-3.737	0.000	Down
CL	CL 72:6_CL 18:0_18:0_16:1_20:5	-3.733	0.000	Down
PE	PE 36:3_PE 18:1_18:2,	-3.722	0.000	Down
PE	PE O-32:1_PE O-16:1_16:0	-3.716	0.000	Down
PE	PE 36:2	-3.713	0.000	Down
FA	FA 16:0	-3.704	0.000	Down
FA	FA 34:0	-3.698	0.000	Down
FA	FA 18:0	-3.686	0.000	Down
PE	PE O-34:2_PE O-16:1_18:1	-3.670	0.000	Down
SL	SL 12:2;O_34:0;O	-3.664	0.000	Down
PC	PC 15:0_18:1(d7)	-3.625	0.000	Down
PG	PG 28:0_8:0	-3.616	0.000	Down
PC	PC 34:2_PC 16:0_18:2	-3.615	0.000	Down
PE	PE 36:3_PE 18:1_18:2	-3.606	0.000	Down
SM	SM 37:1;2O	-3.602	0.013	Down
PS	PS 22:5_22:6;4O	-3.592	0.000	Down
DG	DG 48:11	-3.584	0.023	Down
PI	PI O-11:0_28:6	-3.582	0.000	Down
HexCer	HexCer 16:0;2O_24:1	-3.568	0.000	Down
FA	FA 22:6;4O	-3.564	0.000	Down
MAE	NAE 19:4	-3.559	0.000	Down
SHexCer	SHexCer 16:3;2O_22:3	-3.552	0.048	Down
PE	PE P-34:2_PE P-16:0_18:2	-3.544	0.000	Down
FA	FA 17:0	-3.544	0.000	Down
FA	FA 32:0	-3.531	0.000	Down
PC	PC 36:4_PC 18:2_18:2	-3.530	0.000	Down
FA	FA 18:3;4O	-3.524	0.000	Down
SM	SM 34:2;2O_SM 19:0;2O_15:2	-3.518	0.006	Down
PE	PE 36:2_PE 18:0_18:2	-3.516	0.000	Down
PE	PE-Cer 13:1;2O_30:1	-3.515	0.000	Down
FA	FA 30:0	-3.509	0.000	Down
FA	FA 25:0	-3.507	0.000	Down
SL	SL 16:3;O_36:1;O	-3.505	0.000	Down
PE	PE 36:3	-3.503	0.000	Down
FA	FA 22:0	-3.485	0.000	Down
HBMP	HBMP 22:2_12:0_12:0	-3.483	0.000	Down

PE	PE 34:1_PE 16:0_18:1,	-3.456	0.000	Down
PI	PI 34:1,	-3.455	0.000	Down
SM	SMGDG O-9:0_26:1	-3.444	0.000	Down
PE	PE O-36:3_PE O-18:2_18:1	-3.440	0.000	Down
PI	PI-Cer 13:1;2O_32:7;O	-3.432	0.000	Down
Cer	Cer 12:1;2O_26:0	-3.418	0.026	Down
DG	DG 52:9	-3.415	0.002	Down
FA	FA 29:0	-3.414	0.000	Down
ST	ST 24:1;O4;G_16:2;1O	-3.409	0.000	Down
Cer	CerP 24:2;2O_28:3	-3.409	0.000	Down
FA	FA 26:1;1O	-3.409	0.000	Down
PE	PE 34:1_PE 16:0_18:1	-3.406	0.000	Down
FA	FA 31:0	-3.402	0.000	Down
FA	FA 19:4;1O	-3.397	0.000	Down
FA	FA 20:3;4O	-3.395	0.000	Down
HexCer	HexCer 16:1;3O_26:7;(2OH)	-3.393	0.000	Down
FA	FA 20:0	-3.378	0.000	Down
CL	CL 14:1_22:6_26:0_28:0	-3.369	0.000	Down
FA	FA 14:0	-3.365	0.000	Down
PC	PC 36:3	-3.351	0.000	Down
LPA	LPA 28:2	-3.348	0.000	Down
PE	PE O-18:3_22:5;4O	-3.344	0.000	Down
PI	PI 17:0_22:3;2O	-3.325	0.000	Down
Cer	Cer 12:1;3O_31:0;(2OH)	-3.297	0.000	Down
PI	PI 36:0	-3.297	0.000	Down
PC	PC O-39:8	-3.290	0.001	Down
FA	FA 26:0	-3.286	0.000	Down
OxFA	OxFA 18:0;(2OH)	-3.286	0.000	Down
FA	FA 36:5	-3.285	0.000	Down
FA	FA 24:0	-3.276	0.000	Down
PC	PC O-10:0_22:3;4O	-3.275	0.000	Down
FA	FA 33:0	-3.270	0.000	Down
FA	FA 23:0	-3.261	0.000	Down
PI	PI 34:0	-3.249	0.000	Down
PE	PE-Cer 12:1;2O_16:1	-3.234	0.000	Down
PE	PE O-34:3_PE O-16:1_18:2	-3.233	0.000	Down
FA	FA 28:0	-3.229	0.000	Down
SM	SM 42:2;2O	-3.228	0.000	Down
SL	SL 13:2;O_32:2;O	-3.216	0.000	Down
FA	FA 27:0	-3.214	0.000	Down
SL	SL 13:2;O_36:5;O	-3.210	0.000	Down
TG	TG 49:2;1O_TG 16:0_16:0_17:2;1O	-3.202	0.000	Down
SHexCer	SHexCer 39:0;2O	-3.196	0.000	Down
PC	PC O-12:0_22:5;4O	-3.178	0.000	Down
PI	PI-Cer 36:2;3O	-3.154	0.000	Down
NAGly	NAGly 17:0;O	-3.145	0.000	Down
FA	FA 28:1;2O	-3.139	0.000	Down

PG	PG O-15:0_28:0	-3.136	0.000	Down
PE	PE 34:0	-3.135	0.000	Down
PE	PE 36:2_PE 18:1_18:1,	-3.041	0.000	Down
PC	PC 32:0	-3.033	0.000	Down
CL	CL 12:0_22:6_28:0_28:0	-3.031	0.000	Down
PE	PE 38:5	-3.021	0.000	Down
TG	TG 49:2_TG 16:0_16:1_17:1	-3.019	0.000	Down
PE	PEtOH 26:2_PEtOH 13:1_13:1	-3.014	0.000	Down
SM	SM 33:1;2O	-3.012	0.026	Down
PE	PE 36:3_PE 18:0_18:3	-3.009	0.000	Down
PMeOH	PMeOH 28:7_28:7	-3.009	0.000	Down
PC	PC 34:1_PC 16:0_18:1	-2.962	0.000	Down
FA	FA 22:6	-2.959	0.000	Down
SM	SM 12:0;2O_27:0	-2.957	0.000	Down
HBMP	HBMP 20:1_12:0_12:0	-2.938	0.000	Down
PI	PI 36:2,	-2.924	0.000	Down
TG	TG 46:0_TG 14:0_16:0_16:0	-2.922	0.000	Down
FA	FA 18:1	-2.911	0.000	Down
PS	PS 36:2	-2.897	0.009	Down
PC	PC O-39:10	-2.877	0.000	Down
PI	PI-Cer 13:2;2O_22:6;O	-2.875	0.000	Down
FA	FA 22:5;4O	-2.866	0.000	Down
PC	PC 34:1	-2.851	0.000	Down
Cer	Cer 12:2;2O_42:10;2O	-2.825	0.002	Down
PC	PC O-37:9	-2.810	0.000	Down
SM	SM 40:1;2O	-2.810	0.002	Down
TG	TG 46:1_TG 14:0_16:0_16:1	-2.809	0.000	Down
PI	PI 24:0_18:2;3O	-2.799	0.000	Down
TG	TG 46:1_TG 14:0_14:0_18:1	-2.787	0.000	Down
TG	TG 44:0_TG 14:0_14:0_16:0	-2.766	0.000	Down
PC	PC 40:6	-2.756	0.047	Down
TG	TG 44:1_TG 10:0_16:0_18:1	-2.755	0.000	Down
DG	DG 50:9	-2.746	0.005	Down
TG	TG 43:0_TG 12:0_15:0_16:0	-2.742	0.000	Down
FA	FA 28:7	-2.739	0.000	Down
SM	SM 32:1;2O_SM 17:0;2O_15:1	-2.730	0.000	Down
PE	PE P-38:5_PE P-18:1_20:4	-2.712	0.001	Down
TG	TG 49:2_TG 15:0_16:1_18:1	-2.695	0.000	Down
TG	TG 44:0_TG 14:0_14:0_16:0,	-2.691	0.000	Down
ST	ST 29:1;O;Hex;FA 15:2	-2.686	0.000	Down
PC	PC 36:1_PC 18:0_18:1	-2.682	0.002	Down
TG	TG 48:0_TG 16:0_16:0_16:0,	-2.671	0.000	Down
LNAPS	LNAPS 14:0_N-28:0	-2.660	0.000	Down
TG	TG 47:1_TG 15:0_16:0_16:1	-2.645	0.000	Down
PE	PE 40:5	-2.632	0.000	Down
SM	SM 38:1;2O_SM 18:1;2O_20:0	-2.617	0.020	Down
TG	TG 56:0;1O_TG 22:0_22:0_12:0;1O	-2.578	0.000	Down

PC	PC 39:2	-2.568	0.029	Down
TG	TG O-41:0_TG O-11:0_14:0_16:0	-2.559	0.000	Down
TG	TG 36:0_TG 10:0_12:0_14:0,	-2.552	0.000	Down
PE	PE O-22:4_22:6;3O	-2.544	0.000	Down
TG	TG 43:0_TG 13:0_14:0_16:0	-2.538	0.000	Down
TG	TG 48:2_TG 16:0_16:1_16:1	-2.536	0.000	Down
PI	PI 18:0_28:6	-2.530	0.000	Down
TG	TG O-52:1_TG O-19:1_16:0_17:0	-2.511	0.000	Down
PE	PE 38:4_PE 18:0_20:4,	-2.507	0.029	Down
HBMP	HBMP 22:3_12:0_12:0	-2.506	0.000	Down
TG	TG 46:0_TG 14:0_16:0_16:0,	-2.504	0.000	Down
SM	SM 34:0;2O	-2.483	0.001	Down
HexCer	HexCer 18:0;2O_18:5	-2.479	0.000	Down
PE	PE O-38:6_PE O-18:2_20:4	-2.477	0.002	Down
TG	TG 40:0_TG 12:0_12:0_16:0	-2.474	0.000	Down
SL	SL 16:3;O_30:8	-2.469	0.001	Down
TG	TG 49:0_TG 15:0_17:0_17:0	-2.460	0.000	Down
PC	PC 32:0_PC 16:0_16:0	-2.456	0.000	Down
TG	TG 47:0_TG 15:0_16:0_16:0,	-2.446	0.000	Down
TG	TG 48:0_TG 16:0_16:0_16:0	-2.439	0.000	Down
FA	FA 22:0;4O	-2.436	0.001	Down
PE	PE O-36:2_PE O-18:1_18:1	-2.436	0.000	Down
PE	PE 34:0_PE 16:0_18:0	-2.424	0.000	Down
SHexCer	SHexCer 38:3;3O	-2.422	0.000	Down
NAGly	NAGlySer 22:6_20:1	-2.419	0.000	Down
TG	TG 52:1;3O_TG 17:0_17:0_18:1;3O	-2.387	0.000	Down
TG	TG 36:0_TG 10:0_12:0_14:0	-2.383	0.000	Down
OxFA	OxFA 18:2;(2OH)	-2.382	0.000	Down
SM	SM 40:1;2O,	-2.380	0.000	Down
TG	TG O-54:1_TG O-18:0_18:0_18:1	-2.369	0.000	Down
SM	SM 13:1;2O_28:1	-2.367	0.000	Down
TG	TG 45:1_TG 14:0_15:0_16:1	-2.361	0.000	Down
TG	TG 45:0_TG 15:0_15:0_15:0	-2.354	0.000	Down
PC	PC 36:3_PC 18:1_18:2	-2.351	0.001	Down
PC	PC O-14:0_22:5;3O	-2.317	0.000	Down
TG	TG 58:0_TG 16:0_16:0_26:0	-2.316	0.000	Down
SHexCer	SHexCer 35:0;3O	-2.312	0.000	Down
TG	TG 56:0_TG 15:0_16:0_25:0	-2.308	0.000	Down
PE	PE 38:3	-2.302	0.001	Down
TG	TG 48:1_TG 14:0_16:0_18:1	-2.299	0.000	Down
PE	PE O-24:5_16:0	-2.285	0.000	Down
TG	TG 54:0_TG 18:0_18:0_18:0	-2.283	0.000	Down
TG	TG 42:0_TG 12:0_14:0_16:0,	-2.283	0.000	Down
SM	SM 36:0;2O_SM 24:0;2O_12:0	-2.280	0.020	Down
FA	FA 18:1;O	-2.280	0.000	Down
TG	TG 40:0_TG 10:0_14:0_16:0	-2.280	0.000	Down
TG	TG 49:0_TG 16:0_16:0_17:0	-2.275	0.000	Down

TG	TG 42:0_TG 12:0_14:0_16:0	-2.274	0.000	Down
TG	TG 38:0_TG 8:0_14:0_16:0	-2.273	0.000	Down
TG	TG O-57:1_TG O-19:0_16:1_22:0	-2.269	0.000	Down
PS	PS 36:2,	-2.266	0.000	Down
PE	PE 36:2_PE 18:1_18:1	-2.264	0.000	Down
PC	PC 34:0_PC 16:0_18:0	-2.254	0.000	Down
SM	SMGDG O-21:4_28:7	-2.249	0.000	Down
TG	TG 38:1_TG 10:0_10:0_18:1	-2.247	0.000	Down
TG	TG 42:2_TG 10:0_14:1_18:1	-2.234	0.000	Down
NAGly	NAGlySer 22:6_21:4	-2.215	0.001	Down
SM	SM 13:1;2O_28:2	-2.211	0.000	Down
TG	TG 38:0_TG 12:0_12:0_14:0	-2.210	0.000	Down
TG	TG 38:1_TG 10:0_14:0_14:1	-2.202	0.000	Down
PC	PC O-18:0_18:1;1O	-2.196	0.000	Down
PE	PE-Cer 17:1;2O_36:8;O	-2.174	0.000	Down
DG	DG 44:6	-2.169	0.012	Down
PI	PI 18:0_28:5	-2.169	0.000	Down
TG	TG 48:4_TG 14:0_16:1_18:3	-2.168	0.000	Down
LPE	LPE 18:1,	-2.166	0.000	Down
TG	TG 38:1;1O_TG 8:0_16:0_14:1;1O	-2.132	0.000	Down
ST	ST 24:1;O4;T_21:1	-2.128	0.000	Down
SHexCer	SHexCer 45:4;3O	-2.098	0.000	Down
TG	TG 47:0_TG 15:0_16:0_16:0	-2.086	0.000	Down
PC	PC O-14:1_24:0;1O	-2.083	0.000	Down
DMPE	DMPE 17:0_22:5	-2.080	0.000	Down
SM	SM 12:0;2O_25:0	-2.078	0.000	Down
PE	PE P-38:4_PE P-18:0_20:4	-2.066	0.007	Down
TG	TG 55:0_TG 15:0_16:0_24:0	-2.042	0.000	Down
DG	DGDG 17:1_22:6	-2.038	0.000	Down
TG	TG 42:1_TG 10:0_14:0_18:1	-2.026	0.000	Down
TG	TG 40:1_TG 8:0_16:0_16:1	-2.007	0.000	Down
PI	PI 36:1_PI 18:0_18:1	-1.994	0.000	Down
PS	PS 36:2_PS 18:0_18:2,	-1.992	0.000	Down
PI	PI 20:5_24:0;1O	-1.989	0.000	Down
FA	FA 20:0;4O	-1.982	0.000	Down
TG	TG 55:2_TG 18:0_18:1_19:1	-1.977	0.000	Down
SM	SM 25:3;2O(FA 20:5)	-1.969	0.000	Down
PA	PA 15:0_28:7	-1.967	0.001	Down
PE	PE O-36:5_PE O-16:1_20:4	-1.967	0.005	Down
PE	PE 36:1_PE 18:0_18:1	-1.961	0.000	Down
TG	TG 49:0_TG 16:0_16:0_17:0,	-1.949	0.000	Down
TG	TG 38:1_TG 10:0_10:0_18:1,	-1.939	0.000	Down
PS	PS 22:6_22:6	-1.920	0.001	Down
FA	FA 18:1;2O	-1.917	0.000	Down
PC	PC O-12:0_17:2;2O	-1.916	0.012	Down
TG	TG 40:2_TG 12:0_14:1_14:1	-1.914	0.000	Down
TG	TG O-55:1_TG O-19:1_18:0_18:0	-1.913	0.000	Down

TG	TG 47:1;1O_TG 16:0_16:0_15:1;1O	-1.910	0.000	Down
PI	PI O-13:1_26:7	-1.905	0.000	Down
Cer	CerP 16:1;2O_28:3	-1.902	0.000	Down
PE	PE 36:2;O_PE 18:0_18:2;O	-1.900	0.002	Down
TG	TG 48:2_TG 14:0_16:1_18:1	-1.887	0.000	Down
PA	PA 23:0_28:7	-1.864	0.000	Down
PI	PI 16:0_28:5	-1.863	0.000	Down
PE	PE O-26:5_16:0	-1.858	0.000	Down
TG	TG 53:0_TG 14:0_16:0_23:0	-1.855	0.000	Down
DG	DGGA 22:0_22:6	-1.845	0.000	Down
SHexCer	SHexCer 43:3;3O	-1.835	0.000	Down
PI	PI 24:0_22:6;4O	-1.822	0.000	Down
TG	TG 51:0_TG 16:0_17:0_18:0	-1.808	0.000	Down
MG	MG 18:0	-1.782	0.004	Down
PE	PE-Cer 12:1;2O_32:0	-1.782	0.000	Down
PI	PI 26:0_17:0;2O	-1.782	0.000	Down
PG	PG 18:2_18:1;1O	-1.780	0.001	Down
TG	TG 51:0_TG 17:0_17:0_17:0	-1.777	0.000	Down
PI	PI 24:0_18:1;4O	-1.774	0.000	Down
HexCer	HexCer 20:2;2O_20:5	-1.767	0.001	Down
SM	SM 41:0;3O	-1.762	0.000	Down
ST	ST 29:1;O;Hex;FA 13:0	-1.744	0.000	Down
PE	PE P-36:4_PE P-16:0_20:4	-1.743	0.003	Down
FA	FA 15:0	-1.712	0.000	Down
TG	TG 50:0_TG 16:0_16:0_18:0,	-1.705	0.000	Down
PE	PE 38:5_PE 18:1_20:4	-1.693	0.027	Down
CL	CL 15:0_22:5_28:0_28:0	-1.693	0.014	Down
TG	TG 36:0_TG 12:0_12:0_12:0	-1.691	0.000	Down
TG	TG 49:1_TG 16:0_16:0_17:1	-1.679	0.000	Down
Cer	Cer 16:3;2O_30:3	-1.638	0.000	Down
PE	PE O-26:5_18:2	-1.625	0.003	Down
SM	SM 12:1;2O_29:0	-1.619	0.000	Down
LPE	LPE 18:1	-1.592	0.000	Down
SM	SMGDG O-17:0_28:5	-1.586	0.000	Down
TG	TG 50:0_TG 16:0_16:0_18:0	-1.583	0.000	Down
PI	PI 16:0_22:6;4O	-1.578	0.000	Down
PA	PA 17:0_28:6	-1.559	0.003	Down
PE	PE 36:1_PE 18:0_18:1,	-1.550	0.000	Down
TG	TG 54:0_TG 18:0_18:0_18:0,	-1.546	0.000	Down
TG	TG 56:1_TG 16:0_22:0_18:1	-1.541	0.000	Down
TG	TG 38:0_TG 8:0_12:0_18:0	-1.533	0.000	Down
TG	TG 8:0_9:0_22:1	-1.525	0.000	Down
PE	PE O-19:0_28:6	-1.476	0.030	Down
PC	PC O-12:0_22:3;2O	-1.474	0.003	Down
HexCer	HexCer 16:0;2O_30:4;O	-1.445	0.000	Down
PE	PE 20:4_22:5	-1.436	0.002	Down
SM	SM 39:0;3O	-1.383	0.002	Down

PA	PA 21:0_28:6	-1.365	0.021	Down
TG	TG 48:3_TG 14:0_16:1_18:2,	-1.329	0.001	Down
TG	TG 50:2_TG 16:0_16:1_18:1,	-1.314	0.000	Down
PS	PS 36:1_PS 18:0_18:1,	-1.256	0.001	Down
TG	TG O-49:1_TG O-17:1_14:0_18:0	-1.234	0.001	Down
TG	TG 49:3;1O_TG 16:0_16:0_17:3;1O	-1.210	0.001	Down
TG	TG 34:0_TG 8:0_12:0_14:0	-1.207	0.001	Down
HBMP	HBMP 13:1_12:0_13:1	-1.184	0.001	Down
TG	TG 52:0_TG 16:0_18:0_18:0	-1.182	0.002	Down
HexCer	HexCer 16:0;2O_24:6;O	-1.173	0.002	Down
PE	PE O-13:0_28:7	-1.164	0.003	Down
TG	TG O-50:1_TG O-16:0_16:0_18:1	-1.157	0.001	Down
TG	TG 48:3_TG 12:0_18:1_18:2	-1.155	0.002	Down
TG	TG 50:5_TG 18:1_16:2_16:2	-1.145	0.003	Down
TG	TG 48:3_TG 14:0_16:1_18:2	-1.115	0.003	Down
FA	FA 21:0	-1.106	0.001	Down
TG	TG 55:3_TG 18:1_18:1_19:1	-1.090	0.002	Down
TG	TG 43:3;1O_TG 10:0_18:1_15:2;1O	-1.075	0.012	Down
TG	TG 52:0_TG 16:0_18:0_18:0,	-1.059	0.002	Down
TG	TG 50:1_TG 16:0_16:0_18:1,	-1.042	0.001	Down
PA	PA 17:0_28:7	-1.017	0.004	Down
TG	TG 43:1;1O_TG 10:0_16:0_17:1;1O	-1.009	0.006	Down
SM	SM 12:1;2O_20:0	1.040	0.034	Up
TG	TG 54:3;1O_TG 18:1_18:1_18:1;1O	1.195	0.030	Up
TG	TG O-53:6_TG O-15:4_19:1_19:1	1.486	0.040	Up
FA	FA 42:10	1.660	0.000	Up
LPE	LPE 18:0,	1.920	0.000	Up
Cer	Cer 12:0;2O_19:0;(2OH)	2.162	0.000	Up
SL	SL 13:1;O_34:5	2.179	0.050	Up
LPE	LPE O-18:1	2.221	0.001	Up
TG	TG 54:4_TG 18:1_18:1_18:2	2.244	0.027	Up
LPE	LPE O-16:1,	2.324	0.000	Up
FA	FA 44:10	2.534	0.000	Up
Cer	Cer 12:1;2O_24:3;(2OH)	3.071	0.000	Up
