# Supplementary material for: Untargeted lipidomics reveals unique lipid signatures of extracellular vesicles from porcine colostrum and milk
Source: PLoS One. 2025 Feb 13;20(2):e0313683. doi: 10.1371/journal.pone.0313683 (PMC11825007; doi:10.1371/journal.pone.0313683)
Supplement: Attachment — Submitted filename: Bondoc OL review - PONE-D-24-15462 June 2024.pdf [file pone.0313683.s009.pdf]

PLOS ONE
PONE-D-24-15462

Untargeted lipidomics reveals unique lipid signatures of extracellular vesicles from porcine colostrum and milk.
Rafaela Furioso Ferreira, Morteza H. Ghaffari, Fabricio Ceciliani, Manuela Fontana, Donatella Caruso, Matteo Audano, Giovanni Savoini, Alessandro Agazzi, Vladimir Mrljak, and Helga Sauerwein

Reviewer's comments:

Using the swine model, the manuscript "PONE-D-24-15462" currently under review showed that although the lipid composition of milk extracellular vesicles (EV) was not affected by the maternal diet (i.e., different ratios of ω-6:ω-3 fatty acids in the gestation and lactation diets of sows), distinct extracellular vesicle lipidomic profile were found in colostrum compared to mature milk collected at 7 and 14 days after parturition.

The authors used ultracentrifugation coupled with size exclusion chromatography to isolate EV, nanoparticle tracking analysis and transmission electron microscopy to characterize EV, and assessment of EV markers via Western blotting to assess the EV markers, and liquid chromatography–quadrupole time-of-flight mass spectrometry approach to determine the lipodome.

While it is currently not possible to produce completely pure EV isolates, the manuscript is an important development research to provide a better understanding of the use of porcine milk EV as drug carriers (as "natural liposomes") in a potential natural drug delivery system.

The manuscript was generally well-written although the number of tables and figures may be reduced. Some revisions are suggested as follows:

L48-49, write: … multiple cellular sources (12), MFG are ... Instead of multiple cellular sources, (12) MFG are …

L121, write: … (48) ... Instead of … (47),

L288, write: … main classes (Figure 2) … Instead of … main classes (Fig 2) …

L304, write: … (Supplemental Table S1) … Instead of … (Supplemental Table S4) …

L307, write: … (Supplemental Table S2) … Instead of … (Supplemental Table S5) …

L325, write: … and uptake (37, 38, 72). Instead of … and uptake (37, 38; 72).

L343, please insert reference number in parenthesis Instead of … only one study () has examined …

L357, write: … have different lipid profiles ... Instead of … have different profiles ...

L369-370, insert paragraph to discuss/explain results related to PC (phosphatidylcholine)

L454-679, please check/correct all entries under References following standard journal format and font type (L481-490, L585-599, and L607-608) required by PLOS One.

L701-704, please indicate what A and B charts are all about.
